# Combining molecular characteristics and therapeutic analysis of PDOs predict clinical responses and guide PDAC personalized treatment

**DOI:** 10.1186/s13046-025-03332-8

**Published:** 2025-02-26

**Authors:** Peng Li, Minli Huang, Mengyao Li, Gen Li, Yifan Ma, Yong Zhao, Xiaowu Wang, Yongbin Zhang, Changhong Shi

**Affiliations:** 1 Division of Cancer Biology, Laboratory Animal Center, The Fourth Military Medical University, Xi’an, Shaanxi 710032 PR China; 2 Animal Laboratory Center, Guangzhou University of Chinese Medicine, Guangzhou, 510405 PR China; 3 State Key Laboratory of Holistic Integrative Management of Gastrointestinal Cancers, Xi’an, Shaanxi 710032 PR China; 4 Department of Urology, Tangdu Hospital, Fourth Military Medical University, Xi’an, Shaanxi 710032 PR China; 5 Gansu University of Chinese Medicine, Lanzhou, 730030 China; 6 Department of Cardiovascular Surgery, Xijing Hospital, Fourth Military Medical University, Xi’an, 710032 PR China; 7 Division of Cancer Biology, Laboratory Animal Center, Fourth Military Medical University, Xi’an, Shaanxi 710032 China; 8 Animal Experiment Center, Guangzhou University of Chinese Medicine, Guangzhou, 510405 China

**Keywords:** Patient-derived organoids (PDOs), Pancreatic ductal adenocarcinoma (PDAC), Genomic diversity, Personalized treatment, Immunotherapy

## Abstract

**Background:**

The emergence of targeted therapies and immunotherapy has broadened treatment options for patients with pancreatic ductal adenocarcinoma (PDAC). Despite this, traditional drug selection, predominantly relies on tumor markers and clinical staging, has underutilized these drugs due to ignoring patient genomic diversity. Patient-derived organoids (PDOs) and corresponding patient-derived organoid xenograft (PDOX) models offer a way to better understand and address this.

**Methods:**

In this study, we established PDOs and PDOX models from PDAC clinical samples. These models were analyzed using immunohistochemistry, H&E staining, and genomic profiling. Drug screening with 111 FDA-approved drugs was performed on PDOs, and drug responses in PDOs and PDOX models were compared to assess consistency with clinical treatment outcomes. Gene analysis was conducted to explore the molecular mechanisms underlying variations in drug responses. Additionally, by analyzing the sequencing results from various drug-sensitive groups, the identified differential gene-drug metabolism gene UGT1A10 were modulated in PDOs to evaluate its impact on drug efficacy. A co-culture system of PDOs with immune cells was developed to study the efficacy of immunotherapies.

**Results:**

PDOs and matched PDOX models retain the morphological, biological, and genomic characteristics of the primary tumor. Exome sequencing and RNA sequencing confirmed both the consistency and heterogeneity among the PDOs. High-throughput drug screening revealed significant variability in drug sensitivity across different organoids, yet PDOs and PDOX derived from the same patient exhibited a high degree of concordance in response to clinical chemotherapy agents. The gene expression analysis of PDOs with significant differences in drug sensitivity revealed UGT1A10 as a crucial regulator. The knockdown of UGT1A10 notably increased drug sensitivity. Furthermore, immune cells demonstrated specific cytotoxicity towards the organoids, underscoring the potential of the co-culture system for application in tumor immunotherapy.

**Conclusion:**

Our results highlight the necessity for personalized treatment strategies that consider genomic diversity beyond tumor markers, thus validating the utility of PDOs and PDOX models in advancing PDAC research and personalized medicine.

**Supplementary Information:**

The online version contains supplementary material available at 10.1186/s13046-025-03332-8.

## Background

Pancreatic cancer is one of the most aggressive malignancies, with 85% of patients diagnosed at an advanced stage. Surgery remains the primary treatment option, yet the median 5-year survival rate after surgery is modest (10%), with a recurrence rate ranging from 70 to 80% [1]. Pancreatic ductal adenocarcinoma (PDAC) is the most common form, accounting for most 90% of all pancreatic cancer cases [2]. The current first-line postoperative chemotherapy regimens, such as the combination of gemcitabine and nab-paclitaxel, marginally extend median survival by only 2 to 4 months, underscoring an urgent need for novel therapeutic strategies [3].

The advent of next-generation sequencing (NGS) has revolutionized the landscape of PDAC treatment by identifying genomic tumor drivers and potential targeted therapies [4–5]. By integrating bioinformatics, NGS has been used to identify key mutations and pathways in PDAC, facilitating the development of targeted treatments. These treatments target specific molecular markers to inhibit tumor growth and spread, disrupt cellular signaling, and hinder angiogenesis. However, the complexity of these drugs and the insufficiency of tumor marker expression to capture patient diversity have impeded the clinical use of biomarkers for predicting chemotherapy, targeted therapy, and immunotherapy outcomes [2, 6]. There is an urgent need for a preclinical model that can rapidly and accurately screen drugs based on individual genetic profiles to develop personalized, precision medicine.

Patient-derived organoids (PDOs) are three-dimensional (3D) tumor models that well preserve the genetic, morphological, and cellular diversity of the original tissue, offering a significant preclinical tool for cancer personalized research [7]. This 3D culture conditions also facilitate the study of complex interactions between immune and tumor cells, making the assessment of immunotherapies feasible [8]. While previous studies have shown PDOs’ potential in predicting chemotherapy drug sensitivity [9–11], there is still a gap in research integrating chemotherapy, targeted therapies, immunotherapies, gene sequencing, and clinical outcomes through PDO and corresponding xenograft (PDOX) models.

In this study, we established five pancreatic ductal adenocarcinoma PDOs and corresponding four PDOX models. The histological and molecular features of the original tumor tissue have been characterized using morphological analysis and sequencing. The antitumour effects of 111 commonly used PDAC drugs and immunotherapies were assessed. By integrating NGS analysis, high-throughput drug screening, PDOX experiments, and patient clinical outcome data, personalized drug strategies were comprehensively evaluated. We specifically targeted the differential gene - drug metabolism-related gene UDP-glucuronosyltransferase 10 (UGT1A10) in PDOs to explore its potential as a prognostic biomarker for PDAC. Additionally, a PDO-peripheral blood mononuclear cells (PBMC) or engineered chimeric antigen receptor macrophages (CAR-Ms) co-culture model has been established to test the efficacy of immunotherapies. Our ultimate goal was to develop an ideal preclinical model that captures patient heterogeneity in vitro, contributing to the advancement of personalized treatment for patients with PDAC.

## Materials and methods

### Clinical samples

Clinical tumor specimens and peripheral blood mononuclear cells (PBMCs) were collected from patients with PDAC at the Xijing Hospital, Fourth Military Medical University (FMMU), Xi’an, China (Sup Table 1). Informed consent was obtained from all participants, and the study was approved by the Ethics Committee of Xijing Hospital. Each surgically excised tumor specimen was processed as follows: (i) for whole exome sequencing and RNA-Sequence, (ii) for the development of patient-derived organoids (as detailed in the subsequent methods), and (iii) for embedding tissue sections for pathological analysis. Biopsy samples were exclusively used for organoid culture.

### Tumor isolation and patient-derived organoid culture

For surgical specimens, quickly transfer them to a sterile laboratory environment. Subsequently, wash the specimens 2–3 times with PBS, and perform digestion according to the protocol of the human tumor dissociation kit (Miltenyi Biotec, Bergisch Gladbach, Germany). After dissociation, large fragments were removed by filtering the mixture through a 100-micron cell strainer. Cells were counted, embedded in ice-cold Matrigel, and inoculated in 24-well plates. After at least 30 min at 37 °C, Matrigel (Corning Inc., Corning, NY, USA) was polymerized. The PDAC culture medium (Advanced DMEM/F12 (Gibco, Grand Island, NY, USA), 10 nM HEPES (R&D Systems, Minneapolis, MN, USA), 1×GlutaMAX-1 (Gibco, Grand Island, NY, USA), 1× Penicillin/ Streptomycin solution (Gibco, Grand Island, NY, USA), 500 nM A8301 (R&D Systems, Minneapolis, MN, USA), 10 µM Y27632 (Selleckchem, Houston, TX, USA), 1.56 mM N-acetylcysteine (R&D Systems, Minneapolis, MN, USA), 10 nM Nicotinamide (R&D Systems, Minneapolis, MN, USA), 10 ng/ml FGF10 (R&D Systems, Minneapolis, MN, USA), 1×B27 supplement (R&D Systems, Minneapolis, MN, USA), 10 µM Forskolin (R&D Systems, Minneapolis, MN, USA), 30% Wnt3A conditioned medium (R&D Systems, Minneapolis, MN, USA), 4% Noggin conditioned medium (R&D Systems, Minneapolis, MN, USA) was added and refreshed every 2 to 3 days [10]. For those PDOs that have successfully grown and been derived, the organoids are passaged every 1–2 weeks, contingent upon their individual growth rates. Part of the established PDO was cryop-reserved for sequencing using frozen medium (90% FBS + 10% DMSO) in liquid nitrogen.

### Construction of organoid xenografts

NCG (NOD-*Prkdc*^*em26Cd52*^*Il2rg*^*em26Cd22*^/Gpt) mice and male nude mice were purchased from Collective Pharmacology and maintained in specific pathogen-free animal research facilities at the Laboratory Animal Centre of the Fourth Military Medical University (FMMU). The animal experiments for this study were approved by the Ethics Committee for the Protection of Laboratory Animals of the FMMU (No. 20200602).

Following expansion to a sufficient quantity, Matrigel was gently removed using TrypLE dissociation enzyme (Gibco, Grand Island, NY, USA). The cells were then counted, and approximately 5 × 10⁶ cells were mixed with 100 µl of 50% matrigel and complete medium. This mixture was subsequently injected subcutaneously into 6- to 8-week-old NCG mice [12], successfully establishing four PDOX models. The PDOX tissues were collected for pathological evaluation.

### Histology and staining

Tumor tissues and organoids were fixed in 4% paraformaldehyde and sectioned to a thickness of 4 μm. Hematoxylin and eosin (H&E) staining was performed according to standard histological protocols. Immunohistochemistry was performed with anti-CA19-9 antibody (1:500, Abcam, Cambridge, UK), anti-CEA antibody (1:2000, Abcam, Cambridge, UK), anti-Ki67 antibody (1:500, Abcam, Cambridge, UK), anti-PD-L1 antibody (1:200, Abcam, Cambridge, UK), and anti-HER2 antibody (1:1000, Abcam, Cambridge, UK). To quantify the staining, five random fields per specimen were captured at 20× or 40× magnification under a microscope, and the percentage of positively stained cells was quantified using ImageJ software.

### Drug-screening experiment

In our drug-screening experiment, we employed a multi-concentration approach for several common chemotherapeutic agents. The agents included Gemcitabine, 5-Fluorouracil, Cisplatin, and Irinotecan. As presented in the drug concentration table (Sup Table 2), for each drug, we set five distinct concentration levels, labeled from 5 to 1 in descending order of concentration. For example, the concentration of Gemcitabine at level 5 was 7900 ng/mL, gradually decreasing to 12.64 ng/mL at level 1. This gradient of concentrations was also applied to other drugs such as 5-Fluorouracil, Cisplatin, and Irinotecan, with specific concentration values for each level clearly shown in the table. By using these different concentrations, we aimed to comprehensively evaluate the effects of these chemotherapeutic agents on the experimental models, providing a detailed understanding of their potential therapeutic efficacy and dose-response relationships.

For analyzing the synergy score of Paclitaxel and Gemcitabine, organoids were treated with drugs in a constant concentration (Gemcitabine 0-0.5 µM, Paclitaxel 0–1 µM) for 72 h. Synergy scores were calculated by Synergy Finder (https://synergyfinder.fimm.fi). The final synergy scores were interpreted as follows: less than − 10, the interaction between two drugs is likely to be antagonistic; between − 10 and 10, the interaction between two drugs is likely to be additive; and greater than 10, the interaction between the two drugs is likely to be synergistic.

### High-throughput screening

All 111 pharmaceutical compounds used for the in vitro screening were procured from Selleck (Sup Table 3) [13]. These drugs were stored at -80 °C to prevent repeated cycles of freezing and thawing. In the primary in vitro screening assay, a drug concentration of 10 µmol/L was used [14]. Organoids cultured in 24-well plates were harvested and digested using TrypLE dissociation enzyme. Following microscopic confirmation that the majority of cells had been successfully digested into single cells, the cells were collected via centrifugation at 500 g for 5 min. The supernatant was then removed, and the cells were resuspended in 1 mL of culture medium. After passing through a 70-µm filter, the cells were centrifuged once more and resuspended in complete culture medium. They were then plated in 384-well plates with 10% GFR Matrigel at 3000 cells/30 µL medium/well [15]. Following overnight plating, compounds from the libraries were added to the cultures. A treatment with DMSO served as the negative control. After a 72-hour incubation period, CellTiter-Glo 3D Cell Viability Assay reagent (Promega Corp., Madison, WI, USA) was added to each well. Fluorescence measurements were performed at an excitation wavelength of 560 nm and an emission wavelength of 590 nm. To determine the percent difference in reduction between treated and control cells in cytotoxicity/proliferation assays, the following formula was applied [15]:

$$\:\text{I}\text{n}\text{h}\text{i}\text{b}\text{i}\text{t}\text{i}\text{o}\text{n}\:\text{r}\text{a}\text{t}\text{e}=\frac{\text{F}\text{I}\:590\:\text{o}\text{f}\:\text{c}\text{o}\text{m}\text{p}\text{o}\text{u}\text{n}\text{d}\:\text{t}\text{r}\text{e}\text{a}\text{t}\text{e}\text{d}\:\text{w}\text{e}\text{l}\text{l}}{\text{F}\text{I}\:590\:\text{o}\text{f}\:\text{c}\text{o}\text{n}\text{t}\text{r}\text{o}\text{l}\:\text{w}\text{e}\text{l}\text{l}}\times\:100\%$$

Where: FI 590 = Fluorescence Intensity 590 nm.

The average inhibition rate for each group was calculated using Excel to evaluate the drug effects, and GraphPad Prism 9 was used for data visualization.

### UGT1A10 knockdown

The synthesis of UGT1A10-specific siRNA was designed according to the principles of siRNA design. Briefly, the oligonucleotide used was siUGT1A10, 5’-CAUAUGAUCUCUACAGUCA-3’. To exclude non-specific changes in gene expression profiles due to siRNA delivery, a negative control siRNA containing a random sequence oligonucleotide was also included. For transient transfection, siRNA and Lipofectamine 2000 transfection reagent were diluted separately in OPTI-MEM medium. After standing for 5 min, the diluted siRNA and transfection reagent were mixed and gently pipetted 3–5 times to ensure thorough mixing. The mixture was incubated at room temperature for 20 min, and the final siRNA concentration was 100 nM. This mixture was then combined with PDO, and the siRNA/PDO mixture was seeded into low-attachment 24-well plates at a density of 5 × 10^4^ cells/well and cultured for 48 h in pancreatic cancer organoid culture medium. After 48 h of incubation, cells were collected for further qPCR and Western blot analysis to determine relative expression levels.

### Organoid and immune cells co-culture

Peripheral blood mononuclear cells (PBMCs) were isolated from peripheral blood samples using the Ficoll-Paque density gradient centrifugation method [16]. PBMCs or purified CD3 + T-cells were pre-activated with anti-human CD3/CD28 antibodies in T-cell culture medium at a cell concentration ratio of 1:100 [17]. The medium was RPMI 1640 (Lonza, Breda, The Netherlands) with 2 mM L-glutamine/super glutamine (Gibco, Grand Island, NY, USA), 50 mM Hepes buffer (Lonza), 1% penicillin-streptomycin (Life Technologies), 5 mM sodium pyruvate (Gibco), 1% minimum essential medium non-essential amino acids (Gibco, Grand Island, NY, USA) and 10% human AB serum (Invitrogen, Carlsbad, CA, USA). For T cell co-culture, organoids were released from Matrigel using cold PBS and then resuspended in complete culture medium. The resulting suspensions were passed through a 100-µm strainer to eliminate large organoids. Activated T cells or engineered chimeric antigen receptor-expressing macrophages (CAR-Ms) [18] were co-cultured with the organoids an appropriate ratio for a period of 72 h. Bright field images photographs were captured at 24, 48, and 72 h, and supernatants were collected after 72 h to analysis assess cytokine release. All experiments were repeated three times.

### Immunofluorescence staining in co-culture experiments

Before conducting the co-culture experiment, macrophages and PDOs were stained using the Cell Explorer™ Live Cell Tracking Kit(AAT Bioquest, Sunnyvale, California, USA) according to the protocol. Macrophages were labeled with a green fluorescent marker, while PDOs were labeled with a red fluorescent marker to avoid signal interference. During the co-culture, multiple time points were chosen (e.g., 2, 24, 48, and 72 h), and at each time point, cell images were captured using a confocal microscope to observe the interactions and dynamic changes between macrophages and PDOs. Fluorescence images were captured using a fluorescence microscope equipped with FITC (Ex/Em = 490/520 nm), Texas Red (610/20 nm), or PE-Texas Red filters, with 20X magnification to ensure clear visualization of the fluorescent signals of the cells [19].

### Exome sequencing and analysis

DNA samples were extracted, and quality assessed, with a sequencing depth of 300X (Sup Table 4). The DNA was fragmented into 150–350 base pair segments, followed by end repair, addition of an ‘A’ base to the 3’ end, and ligation of double-stranded adapters to generate the library. The library was hybridized with biotin-labeled exome capture probes, enriched using streptavidin-coated magnetic beads, and PCR-amplified. After quality control, sequencing was performed on the Illumina NovaSeq 6000 platform (PE150). SNP and InDel analysis were conducted using Mutect2 for paired samples and population cohorts for non-paired samples. Raw sequencing data were processed to remove adapter contamination, low-quality reads, and ambiguous nucleotides (N), then aligned to the human reference genome GRCh37 using Burrows-Wheeler Aligner (BWA). Duplicate reads were removed, and base quality scores were recalibrated using GATK, incorporating BQSR (Base Quality Score Recalibration) [20].

### RNA-sequencing and analysis

Total RNA was isolated from the samples using TRIzol reagent (Thermo Fisher Sci., Waltham, MA, USA.). mRNA was fragmented and reverse transcribed into cDNA. After end repair, purification, and enrichment, paired-end 150-base pair (PE150) sequencing was performed on the Illumina NovaSeq 6000 platform (LC Bio Technology CO. Ltd, China) according to the standard protocol.

Raw sequencing data were filtered using Cutadapt to remove poor-quality reads. The filtered data were aligned to the reference genome using Hisat2. The alignment results were used for transcript reconstruction and gene expression quantification with StringTie. Gene expression levels of protein-coding genes (mRNA) were statistically analyzed to determine correlations within and between groups, and to identify differentially expressed genes. Gene expression levels were quantified using Fragments Per Kilobase of transcript per Million mapped reads (FPKM). Differentially expressed genes were identified based on a fold change (FC) ≥ 2 and a q-value < 0.05. For multiple group comparisons, genes with a q-value < 0.05 were considered differentially expressed without applying fold change thresholds [21].

### Western blot

The PDAC cell lines AsPC-1 and BxPC-3 were obtained from the American Type Culture Collection (ATCC). The gemcitabine-resistant AsPC-1 (AsPC-1_ GE) subline was established in-house through sustained culture in gemcitabine (Sup Fig. 5A). All cells were maintained in Dulbecco’s modified Eagle’s medium (DMEM) with 10% fetal bovine serum and confirmed mycoplasma-free. Proteins were analyzed using standard protocols. Primary antibodies included anti-UGT1A10 (1:1000, SAB, #42804), anti-p-AKT (1:2000, Cell Signaling, #4060) and anti-AKT (1:1000, Cell Signaling, #4691). Protein bands were quantified by grayscale analysis using ImageJ (v2020).

### Q‑PCR

Total RNA was extracted from cell using TRIzol (Invitrogen). mRNA was used for first-strand cDNA synthesis with a Step One Plus real-time PCR Detection System (Applied Biosystems, Thermo Fisher, Waltham, MA, USA).as per the manufacturer’s instructions. q-PCR was a repeated at least three times independently, with actin as the loading control. Primer sequences are shown in Table S5.

### Statistical analysis

All data were expressed as the mean ± SEM. Differences between groups were analyzed using one-way ANOVA, followed by post-hoc multiple comparisons using the least significant difference (LSD) test. Data were approximately normally distributed, and the variance was homogeneous between groups. Significance was set at *P* < 0.05. Statistical analyses were performed using GraphPad Prism 8.0.2 (RRID: SCR_002798). ns *P* > 0.05, * *P* < 0.05, ** *P* < 0.01, *** *P* < 0.001.

## Results

### Establishment of organoids and xenograft models derived from PDAC patient

**Fig. 1:**
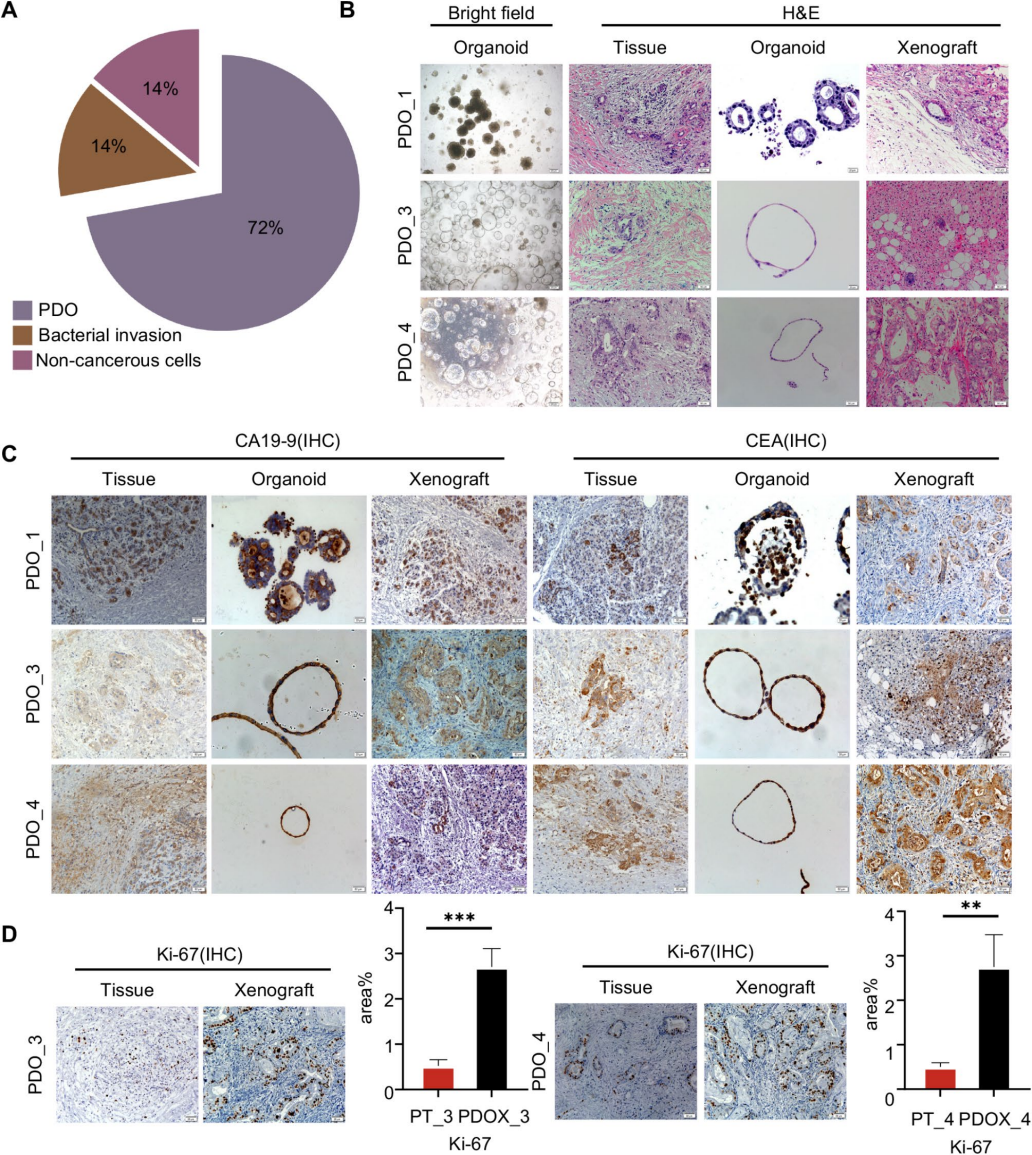
PDOs and corresponding PDOX models maintain the histopathological features of the original tumors. (**A**) Five PDOs (71%) were established from seven PDAC specimens; the other two failed due to bacterial contamination and an insufficient number of tumor cells in the biopsy samples. (**B**) Bright field microscopy images that have been cultured for more than five generations. Scale bar:200 μm. Representative H&E staining images of PDAC tumor tissue with corresponding PDO and PDOX. Scale bar, 50 μm. (**C**) Representative IHC images of paired PDAC tumors, PDO and PDOX for CA19-9 and CEA, Scale bar: 50 μm. (**D**) IHC images of paired PDO_3 and PDO_4 primary tissues and PDOX for Ki-67, Scale bar:50 μm.(*P < 0.05, **P < 0.01, ***P < 0.001)

Fresh PDAC patient specimens were collected post-surgery and tumor cells were cultured as organoids in Matrigel [11]. Five PDOs were successfully established from seven PDAC specimens (5/7, 71%), with two failures due to bacterial infection and insufficient tumor cells in the biopsy (Fig. 1A). After expansion, PDOs were injected subcutaneously into NCG mice, resulting in the establishment of 4 PDOX models. Tumor formation occurred within 2 weeks to 1 month (80%, 4/5), and all PDOs were stably passaged with lumen-like and cystic morphology (Fig. 1B).

To verify whether PDOs and PDOX models retained the histopathological features of the original tumor tissue, we performed H&E staining on the original tissue, PDOs, and PDOX models. Both PDOs and PDOX models maintained the hollow glandular structures characteristic of PDAC (Fig. 1B). Specifically, PDO_1 exhibited cystic structures, while PDO_3 and PDO_4 retained lumen-like features, demonstrating that both models accurately replicate the morphological characteristics of the primary tumor (Fig. 1B).

We further examined the expression characteristics of tumor-specific markers in the tumor tissue, PDOs, and PDOX models. Histological results showed that the expression of carbohydrate antigen CA19-9 and carcinoembryonic antigen CEA was basically consistent in the original tissue, PDOs, and PDOX models [22] (Fig. 1C). However, the expression of Ki-67 was higher in PDOs and PDOX models (Fig. 1D), which may be related to the selection of tumor stem cells during organoid culture.

In summary, we successfully established 5 PDOs and corresponding four PDOX models. These results demonstrated that the histological structure, differentiation state, and morphological heterogeneity of the primary tumor are basically consistent with corresponding PDOs and PDOX models, effectively preserving the histological characteristics and heterogeneity of the primary tumor.

### Preservation of PDO for the mutational landscape of original tissues

Compared to normal cells, cancer cells undergo various genomic alterations in the genome, including single nucleotide variants (SNV), insertions and deletions (InDel), and other types of variations [23]. To characterize the genomic changes of PDO and assess whether PDOs retain the genetic characteristics of the original tissue, we performed whole-exome sequencing on two original tumor tissues and five PDOs.

**Fig. 2:**
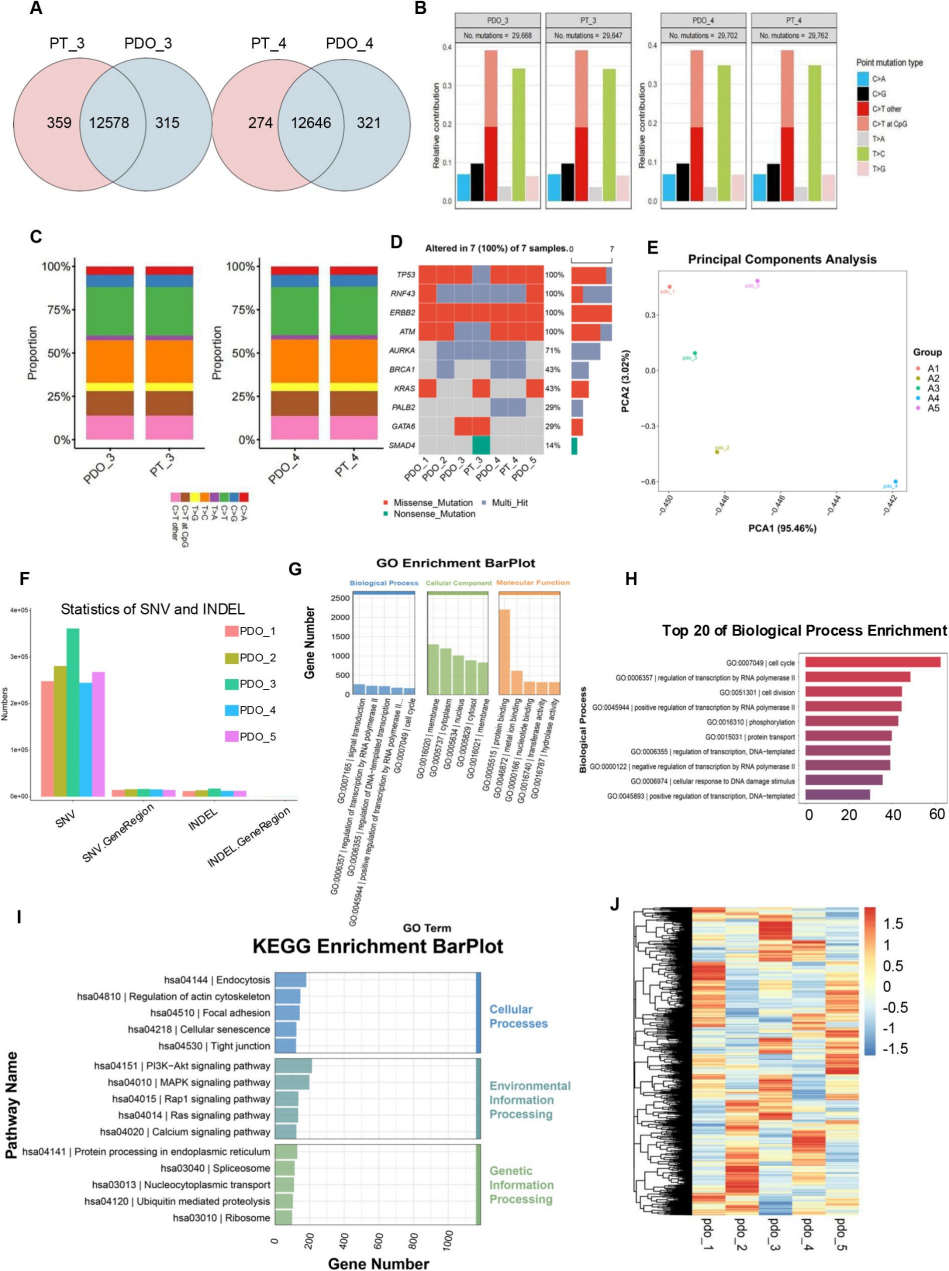
PDOs preserve the mutational landscape of the original tissue while exhibiting consistency and heterogeneity. (**A**) The somatic genomic landscape of PDOs and the corresponding parental tumors. (**B**) The overall distribution of base substitution types identified in both organoid samples and their corresponding tissue samples is presented. (**C**) The proportion of exonic variations identified in each organoid and its corresponding tissue is detailed. (**D**) A table summarizing the gene expression levels of common pancreatic cancer driver genes and drug target genes across five PDOs and two original tissues is provided. (**E**) Comparison of 5 PDOs using a Principal Component Analysis (PCA) chart. (**F**) The number of SNVs and INDELs in the five PDOs were respectively quantified and expressed. (**G**) The GO enrichment analysis of 5 PDOs. (**H**) The 20 most significant biological processes were selected for graphical representation. (**I**) KEGG-enriched barplots of 5 PDOs. (**J**) Hotspot map of differentially expressed genes in 5 PDOs. The dendrogram on the left shows the results of sample cluster analysis, intuitively presenting the similarities and differences among samples. The color bar on the right corresponds to a numerical range from − 1.5 to 1.5, with red indicating higher values and blue indicating lower values

Comparing the whole-exome data of the original tissue with the matched PDO, PDO_3 had a 97.6% (12578/12893) overlap with its matched original tumor tissue PT_3 in terms of gene mutation sites, which was 97.5% (12646/12967) for PDO_4 (Fig. 2A). This indicates that short-term 3D culture in vitro only produced approximately 3% of unnecessary mutations, and PDOs effectively retained the fundamental characteristics of the original tumor at the genomic level.

In the matched PDAC tissues and PDOs, the most common base pair mutations were C > T, while the least common was T > A, consistent with previous reports [24]. In two pairs of PDAC tissues and their corresponding PDOs, the predominant mutational signature observed is the C > T transition, notably at CpG sites (5’-CG-3’). Such mutations are typically indicative of errors in endogenous processes and are associated with DNA methylation damage as well as age-related mechanisms [25]. The PDOs closely replicate the original tumor tissues in terms of both the number and type of base mutations, and their mutational profiles align with those previously reported in the literature [16] (Fig. 2B/C).

We examined the expression levels of driver genes and drug-target genes in five PDOs and two primary tumor tissues, with a focus on TP53, RNF43, ERBB2, ATM, AURKA, and GATA6. The findings indicate that PDO_4 effectively preserved the key mutations present in the primary tumor tissue (PT_4), demonstrating a consistent mutation status between the primary tumor and its corresponding PDO.

Notably, the nonsense mutation of SMAD4 in PT_3 was not directly retained in PDO_3, which may be due to the intra-tumor cell diversity that occurred during the tumor progression [26]. Compared with PDO_3, PDO_4 lacked the GATA6 mutation but had gene mutations in PALB2 and BRCA1 (Fig. 2D), both of which are critical drug target genes [27].

In summary, whole-exome sequencing results show that the gene mutation characteristics between the primary tumor and matched PDO are highly consistent. Short-term culture in vitro only led to a limited number of unnecessary mutations. Organoids retained the mutation profiles of the original tissue, including mutation sites, somatic base substitutions, and mutations in driver genes and drug target genes.

### Consistency and heterogeneity among PDOs

RNA-seq facilitates a comprehensive analysis of gene function and structure, offering vital insights into specific biological processes [28]. Therefore, to further characterize the differences in gene structure and function among PDOs and explore the individual differences among five PDOs, we performed RNA-Seq. Through principal component analysis (PCA) dimensionality reduction of the principal component analysis, the five PDOs were randomly distributed (Fig. 2E).

Compared to normal cells, cancer cells undergo various abnormal changes in the genome, including SNV, InDel, and other types of variations [29]. To investigate the origins of individual variation, we analyzed the SNV and InDel mutations in the coding regions of the five PDOs (Fig. 2F). Our findings revealed that mutations were predominantly situated in intronic regions, in contrast to exonic coding regions. This pattern implies that PDAC development and progression are characterized by significant genomic instability, resulting in the accumulation of mutations. Only a minority of these mutations are located in functionally critical exons and are instrumental in driving tumorigenesis. The majority of mutations are likely to be “passenger” in nature, meaning they do not directly contribute to the initiation or progression of the tumor. The incidence of SNV mutations was higher than that of InDel mutations, with A > G and C > T transitions being the most prevalent base pair changes across the five PDOs. Irrespective of whether the mutations were SNVs or InDels, the PDOs displayed consistency in the quantity, type, and distribution of mutations, with the primary distinction being the increased frequency of SNV mutations (Sup Fig. 2). The variation in mutation frequencies may indicate differing mutational burdens across specific genes within these PDOs, despite their overall consistency in mutational profiles.

The results of GO functional enrichment analysis revealed that the gene products predominantly localized in the cell membrane, nucleus, cytoplasm, and sol-gel of pancreatic cancer cells are actively engaged in performing essential functions, highlighting their integral role in key cellular structures (Fig. 2G). Further analysis indicates that these gene products are critically involved in several fundamental biological processes, including cell cycle regulation, RNA polymerase-mediated transcription, and cell division (Fig. 2H). These findings underscore the active participation of these gene products in processes vital for cell proliferation and functional regulation, potentially offering insights into the pathogenesis and progression of pancreatic cancer.

The KEGG-enriched pathways predominantly involved cell cycle-related pathways, such as the PI3K-AKT, MAPK, Rap1, RAS, and calcium pathways (Fig. 2I). These pathways are well-established for their roles in mediating cell growth, proliferation, survival, and apoptosis, all of which are central to the regulation of the cell cycle and are frequently dysregulated in cancer, including pancreatic cancer. However, when analyzing the differential gene expression profiles of the five PDOs, the clustering heatmap revealed significant differences in gene expression patterns across the samples (Fig. 2J). Despite showing some similarity in overall mutational features and enrichment of cell cycle-related pathways, the distinct gene expression patterns suggest a certain degree of heterogeneity in the molecular mechanisms driving the progression and behavior of each PDO. This heterogeneity likely reflects differences in how individual genes interact with core pathways in each PDO, which may influence their sensitivity to cell cycle regulation and related therapeutic targets. These findings further underscore the molecular complexity of PDAC and highlight the importance of accounting for individual variability in the development of personalized therapeutic strategies.

### Conduct high-throughput drug screening on PDO and validate in corresponding PDOX and patient

**Fig. 3:**
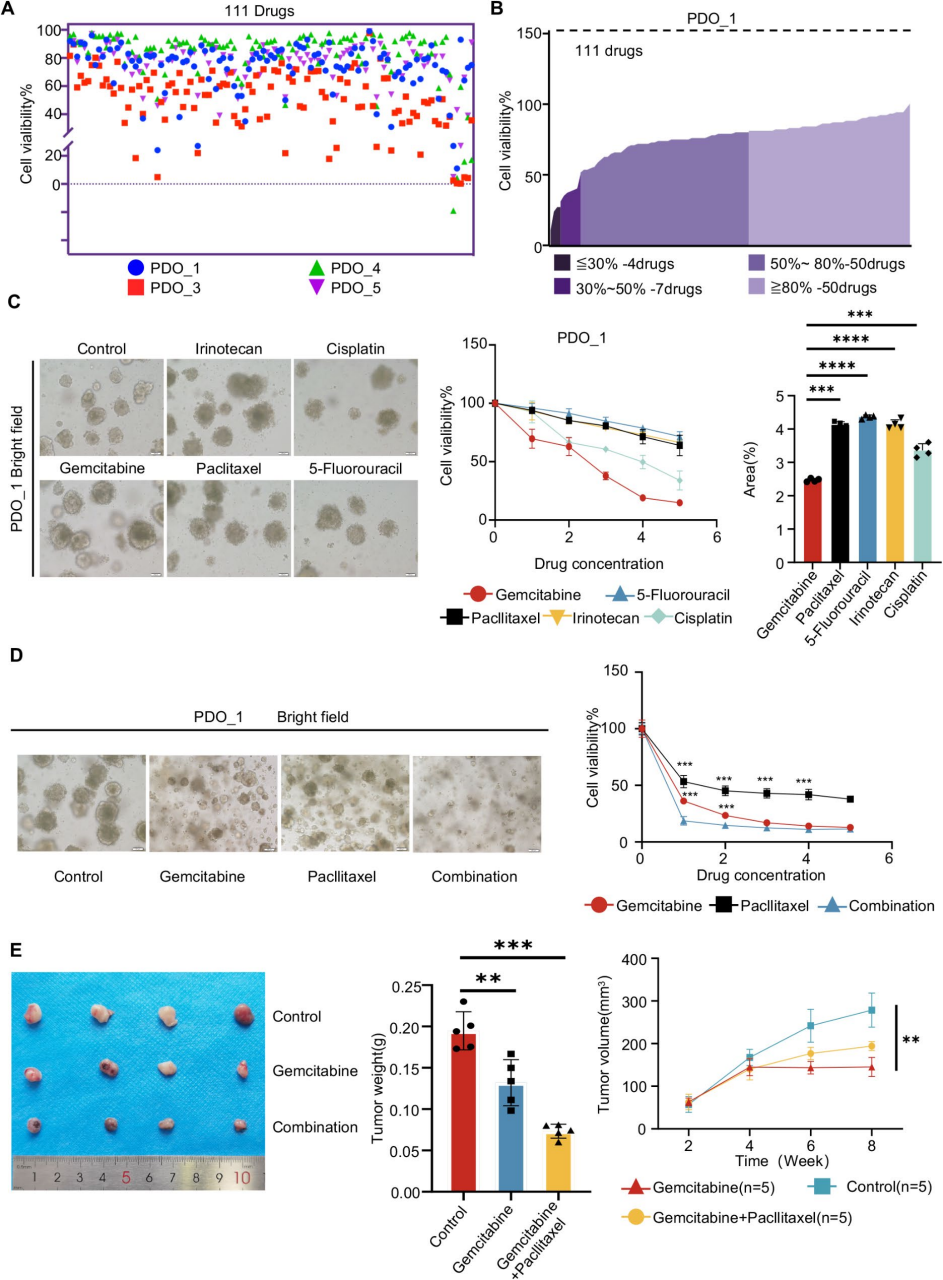
High-throughput drug screening platform utilizing pancreatic cancer organoids. (**A**) Cell viability assessment of 111 first-line chemotherapeutic agents in organoids derived from patients 1-, 3-, 4-, and 5. Different colors stand for different patient-derived organoids. (**B**) Preliminary screening result for PDO_1. (**C**) Representative brightfield microscopy images of PDO_1 cells treated with control, irinotecan, cisplatin, gemcitabine, paclitaxel and 5-Fluorouracil. Scale bars, 100 μm. Summary of chemosensitivity responses for PDO_1 ex vivo to irinotecan, cisplatin, gemcitabine, paclitaxel and 5-Fluorouracil. The results are graphically represented through dose-response curves. The AUC was calculated from the raw dose-response data and is presented as a bar graph for easy comparison and analysis. According to Table S2, to ascertain the Maximum and Minimum Concentrations of each drug, a five-point dose dilution series was established, with 5 representing the Maximum Concentration, 1 representing the Minimum Concentration, and 0 indicating the blank control. (**D**) Gemcitabine, paclitaxel, and their combination therapy were re-administered to PDO_1, with the outcomes visualized through brightfield microscopy and depicted via counting cell number. (**E**) Growth curves of PDO subcutaneous xenografts in NCG mice. The mice were treated with either a vehicle control, gemcitabine alone, or a combination of gemcitabine and paclitaxel, with administrations twice weekly over a 14-day period (*n* = 5). Results are presented as tumor volume (mean ± SD). Representative images and tumor weights of the xenografts in drug/vehicle-treated mice at the end of the experiment are shown

To determine the accuracy of PDOs in reflecting drug sensitivity in patients with PDAC, we assessed the cell viability of 111 first-line chemotherapeutic agents in organoids derived from patients 1, 3, 4, and 5 (Fig. 3A, Sup Table 3). Notably, PDO_1 exhibited a broad drug responsiveness. We subjected PDO_1 to a drug library comprising 111 first-line chemotherapeutic agents, and found that 50 of these drugs achieved a tumor inhibition rate of over 80% (Fig. 3B). This included two drugs that inhibit tumor angiogenesis and proliferation, two drugs that target and inhibit the cellular proteasome, two mTOR inhibitors targeting the PI3K/Akt/mTOR pathway, 11 tyrosine kinase inhibitors targeting the PI3K/AKT and MAPK pathways, nine drugs targeting the ATM gene to block DNA synthesis, inhibit nucleotide metabolism, and induce cell apoptosis, and 3 MEK signaling pathway inhibitors [30].

The results highlighted significant variations in the efficacy of different drugs against the same PDO, indicating that drugs with stronger tumor-killing effects often target pathways enriched in the PDO genetic profile. Therefore, the gene expression patterns of PDOs may provide valuable insights into their drug sensitivity, suggesting that genetic targets could serve as potential indicators of therapeutic responsiveness. Rapid and effective high-throughput screening of these drugs can facilitate informed clinical treatment choices for patients.

Subsequently, we selected first-line pancreatic cancer drugs, including gemcitabine, paclitaxel, 5-Fluorouracil (5-FU), irinotecan, and cisplatin for further drug rescreening of PDO_1 and plotted drug-dose effect curves [2] (Fig. 3C). Drug sensitivity was quantified by calculating the area under the drug-dose curve (AUC) and statistical analysis was performed. The results revealed that PDO_1 was most sensitive to gemcitabine, displaying a concentration-dependent response, while its sensitivity to other drugs was markedly lower (Fig. 3C). Furthermore, the combination of gemcitabine and paclitaxel (One of the common combination treatment strategies for clinical PDAC patients.) yielded a greater tumor cell inhibition rate than either drug alone (Fig. 3D). A synergy analysis of Paclitaxel and Gemcitabine was conducted. The two drugs were applied to organoids within a specific concentration range for 72 h, and then the Synergy Finder was used to calculate the synergy score. According to the criteria [31], the results showed that the synergy score of the two drugs was 10.921, indicating a synergistic effect (Sup Fig. 2). Finally, to verify whether the results of in vitro experiments were significant, we constructed matched PDOX model of PDO_1 and conducted in vivo experiments. The results showed that the tumor volume and weight in the gemcitabine combined with paclitaxel group were significantly different from those in the control and gemcitabine groups (Fig. 3E). In addition, after performing Ki-67 immunohistochemical staining on the PDOX sections of the control, gemcitabine, and combination treatment groups, differences were also found among the groups (Sup Fig. 3). These findings underscore the potential use of PDOs as predictive models for drug sensitivity, thereby informing treatment strategies for patients.

We further validated the drug-sensitive results from PDO_1 in clinical settings. Patient 1 (PT_1), with moderately differentiated PDAC, had a serum CA19-9 level of 70.2U/ml post-surgery. After three months, the patient underwent a 4-month treatment with gemcitabine and nab-paclitaxel over 4 cycles, which reduced the tumor marker to a minimum of 16U/ml, while the maximum value is 27, 9516U/ml (Sup Fig. 4). This suggests the combination therapy effectively suppressed tumor growth. These findings suggested that PDO and matched PDOX can serve as parallel preclinical models to indicate clinical drug use for patients. Analyzing PDOs at the molecular and therapeutic levels can predict clinical responses, guiding treatment decisions.

### Screening for targets and pathways related to drug sensitivity through PDO

**Fig. 4:**
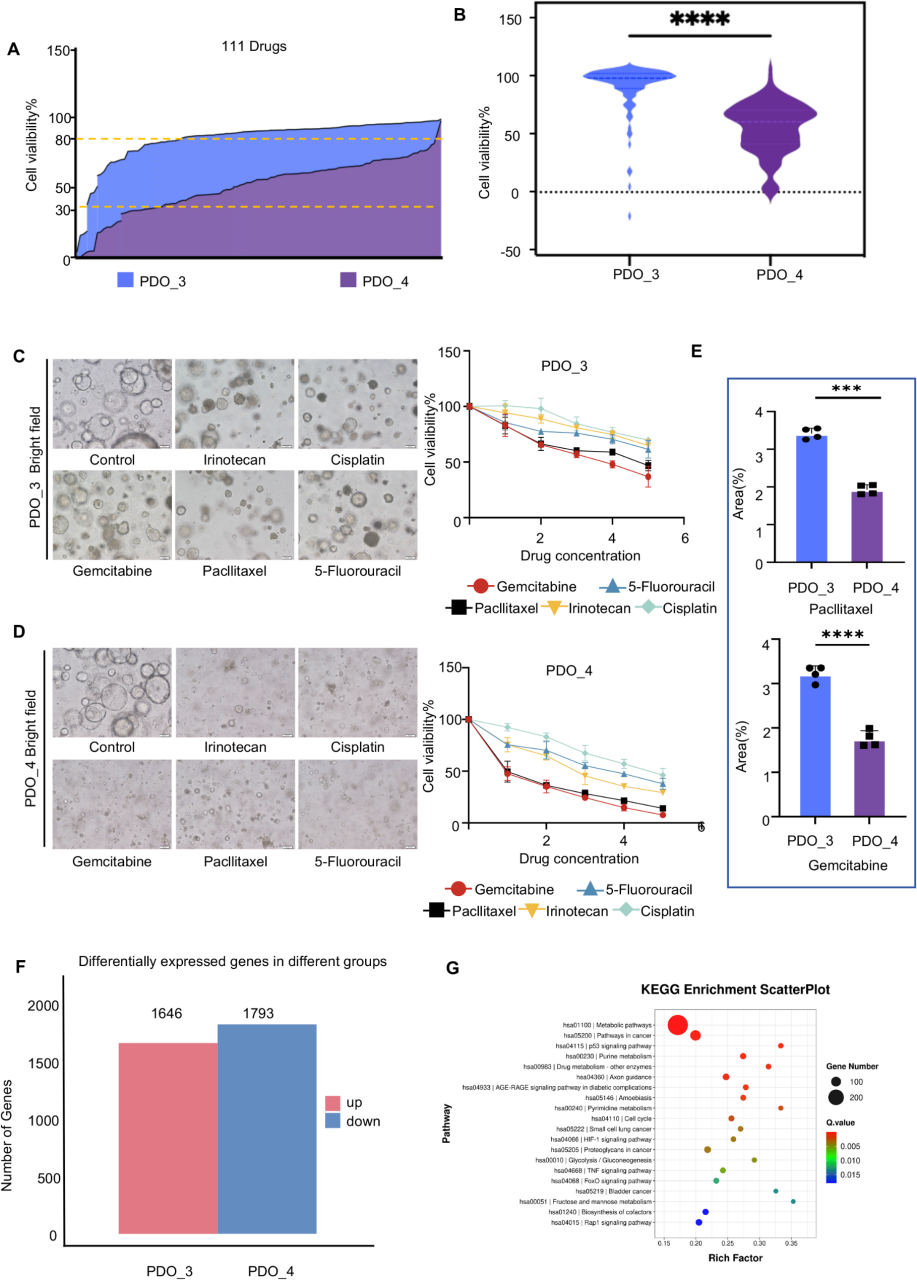
Identifying drug sensitivity-associated targets and pathways using PDOs. (**A**) Preliminary screening result for PDO_3, 4. (**B**) High-throughput drug screening differential analysis of PDO_3, 4. The dendrogram on the left shows the results of gene cluster analysis, presenting the similarities and differences among different genes. The color bar on the right corresponds to the numerical range of gene expression levels from 0.5 to 2.5. The darker the color, the higher the gene expression level. C-D. Representative brightfield(left) and dose-response curves(right) of PDO_3 (**C**), 4 (**D**) exposed to control, irinotecan, cisplatin, gemcitabine, 5-FU and paclitaxel. According to Table S2, to ascertain the Maximum and Minimum Concentrations of each drug, a five-point dose dilution series was established, with 5 representing the Maximum Concentration, 1 representing the Minimum Concentration, and 0 indicating the blank control. scale bars, 100 μm. **E**. Statistical analysis of the AUC for PDO_3, 4 exposed to paclitaxel and gemcitabine Each point indicates the mean value of three replicates (*P < 0.05, **P < 0.01, ***P < 0.001). **F**. Number of differentially expressed genes in PDO_3, 4. G. The KEGG pathway enrichment scatter plot displays the top 20 KEGG metabolic pathways for PDO_3, 4. The size of the circles represents the number of genes enriched in the corresponding pathways, with colors ranging from blue to red indicating decreasing P-values

To further explore the reasons for differences in drug sensitivity among patients, we used a library of 111 pancreatic cancer drugs to perform high-throughput drug sensitivity tests on PDO_3 (drug-resistant) and PDO_4 (drug-sensitive). The results showed significant differences in drug sensitivity of PDO derived from different patients (Fig. 4A-B). Among them, 99 pancreatic cancer drugs achieved a tumor inhibition rate of over 80% for PDO_4. However, only 4 drugs achieved an 80% tumor inhibition rate for PDO_3. To further verify these differences, we choose five clinically common drugs gemcitabine, 5-FU, irinotecan, paclitaxel, and cisplatin for drug rescreening and plotted drug-dose effect curves. Drug sensitivity was quantified by calculating the AUC. The results demonstrated that PDO_4 had good sensitivity to the first-line clinical drugs gemcitabine, paclitaxel, 5-FU, irinotecan, and cisplatin, all of which were significantly better than those of PDO_3 (Fig. 4C-E).

Based on the results, we compared the transcriptome data of those two PDOs. The results showed that the number of differential genes between the two groups was significant. PDO_3 had 1646 mutated genes, while PDO_4 had 1793 mutated genes (Fig. 4F). The KEGG-enriched pathways were mainly related to cell cycle and cell differentiation pathways (Fig. 4G). The substantial differences in gene expression may offer a molecular explanation for the observed disparities in drug sensitivity between PDO_3 and PDO_4, potentially owing to unique genetic and pathway alterations. For instance, specific mutations can influence the efficacy of chemotherapeutic agents, resulting in variations in drug responsiveness.

### The impact of UGT1A10 on drug sensitivity in PDAC patients

**Fig. 5:**
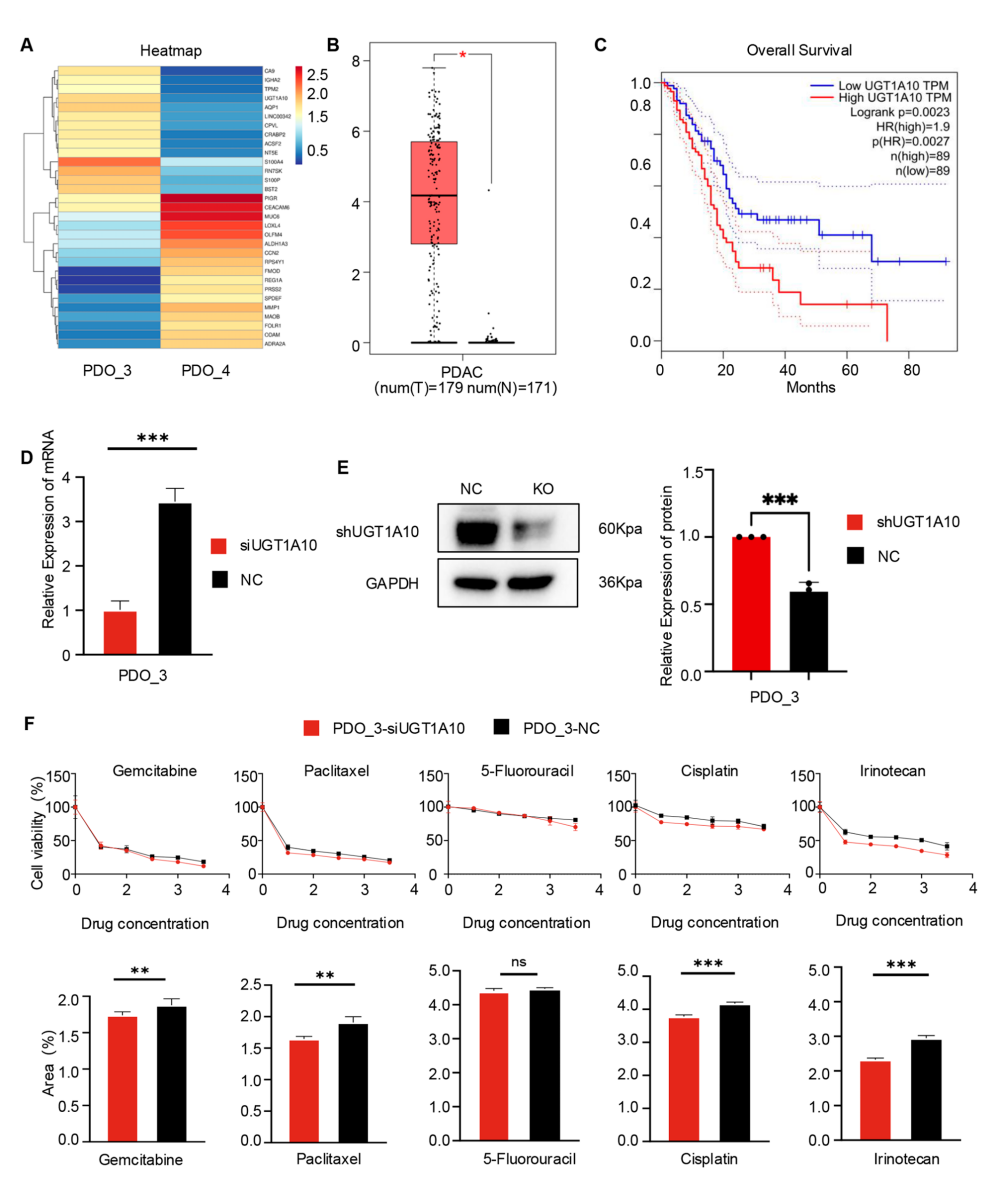
The influence of UGT1A10 on drug sensitivity in patients with PDAC. A heatmap was used to illustrate the significant differential gene expression between PDO_3 and PDO_4. The colors range from red to blue, indicating decreasing P-values. **B**. The box plot demonstrates that UGT1A10 gene expression is elevated in normal tissue compared to that in tumor tissues. **C**. Patients with PDAC exhibiting low UGT1A10 expression had significantly poorer overall survival (OS) compared to those with high UGT1A10 expression. **D**. The mRNA expression levels of UGT1A10 in PDO_3 cells were assessed following treatment with siUGT1A10 or scrambled siRNA (NC). **E**. Protein expression levels and quantitative analysis results for shUGT1A10 in PDO_3 were evaluated following treatment with shUGT1A10 or scrambled shRNA (NC). **F**. Dose-response curves and the corresponding statistical analysis of the AUC for PDO_3 cells exposed to gemcitabine, paclitaxel, 5-Fluorouracil, cisplatin and irinotecan after pretreatment with siUGT1A10 or scrambled siRNA (NC) According to Table S2, to ascertain the Maximum and Minimum Concentrations of each drug, a five-point dose dilution series was established, with 5 representing the Maximum Concentration, 1 representing the Minimum Concentration, and 0 indicating the blank control. (*P < 0.05, **P < 0.01, ***P < 0.001)

We used RNA-seq to screen for the 20 most significantly differentially expressed genes between PDO_3 and PDO_4 (Fig. 5A). Subsequently, these genes were subjected to bioinformatics analysis using the TCGA database. The results showed that UGT1A10 was highly expressed in tumor tissue and significantly correlated with poor prognosis in patients (Fig. 5B-C). Considering the role of UGT1A10 in the metabolism of various chemotherapeutic drugs, its overexpression may accelerate drug clearance or inactivation, thereby reducing therapeutic efficacy [32].

To further verify the impact of this gene on drug sensitivity, we downregulate the expression of the UGT1A10 gene in PDO_3 using lentiviral transduction for testing. PCR and Western Blot results confirmed successful knockdown of UGT1A10, named sh-PDO_3 (Fig. 5D-E). Subsequently, the clinically common drugs gemcitabine, cisplatin, paclitaxel, irinotecan, and 5-FU were used for drug sensitivity testing of PDO_3 and si-UGT1A10. The results demonstrated that better tumor-killing effects was observed in the si-UGT1A10 group for gemcitabine, irinotecan, cisplatin, and paclitaxel, but worse drug sensitivity was detected for 5-FU. Overall, the expression of UGT1A10 affected the drug sensitivity of PDO_3 to commonly used clinical drugs (Fig. 5F). We further established the gemcitabine-resistant PDAC cell line, AsPC-1_GE, through continuous exposure to gemcitabine (Sup Fig. 5A). Western blot analysis revealed that AsPC-1_GE has elevated UGT1A10 expression levels compared to the parental AsPC-1 line (Sup Fig. 5B). In our analysis of the GEO dataset GSE140077 (https://www.ncbi.nlm.nih.gov/geo/query/acc.cgi), we compared the bulk RNA-seq data between three human PDAC cell (CFPAC-1) samples and their corresponding three gemcitabine-resistant counterparts. The volcano plot displays the outcomes of this analysis, with statistical significance indicated by Padj < 0.05 and fold change (LogFC) thresholds of > 1 or < -1. Red points denote upregulated genes, blue points represent downregulated genes, and gray points signify genes without significant differences. The encircled point highlights the upregulated differential gene UGT1A10 (Sup Fig. 6). In addition, we have obtained a series of important findings closely related to the role of UGT1A10 in pancreatic cancer. In the TNM staging system, “T” represents the size of the primary tumor and the extent of local invasion. The larger the value, the larger the tumor or the more extensive invasion range; “N” represents the involvement of regional lymph nodes, with higher values indicating more severe lymph node metastasis; In this staging system, “M” represents distant metastasis, where “M1” indicates the presence of distant metastasis, and “Mx” indicates that the status of distant metastasis cannot be evaluated. Based on this system, we conducted our relevant research. First, the expression level of UGT1A10 was found to exhibit a positive correlation trend with the pathological grade of the corresponding clinical pancreatic cancer (Sup Fig. 7A). When we ranked the clinical information corresponding to PDO according to the severity of the disease from high to low as pT3N1Mx (PDO_3), pT2NxM1 (PDO_1), pT2N0M1 (PDO_2) and pT2N1Mx (PDO_4), we further found that this ranking of disease severity also exhibited a positive correlation trend with the expression of UGT1A10 (Sup Fig. 7B).

In the process of in - depth exploration of the function of UGT1A10, after knocking down UGT1A10 in the organoid PDO_3, qPCR detection revealed that the related drug - resistant genes showed a significant downward trend (Sup Fig. 7C). The KEGG enrichment bar chart intuitively shows that UGT1A10 can participate in the regulation of the drug - resistance mechanism through the PI3K - AKT pathway (Sup Fig. 7D). Compared with the parental AsPC-1 cell line, the gemcitabine - resistant cell line AsPC-1_GE exhibits increased expression of UGT1A10, p-AKT and AKT. In contrast, compared with the negative control group (NC group), the organoids with UGT1A10 knockdown show decreased expression of p-AKT and AKT (Sup Fig. 7E). The above - mentioned series of results comprehensively and deeply demonstrate the crucial role of UGT1A10 in the pathological grade and drug - resistance mechanism of pancreatic cancer.

### Evaluating the immunotherapy efficacy of pembrolizumab and CAR-Ms through PDO

**Fig. 6:**
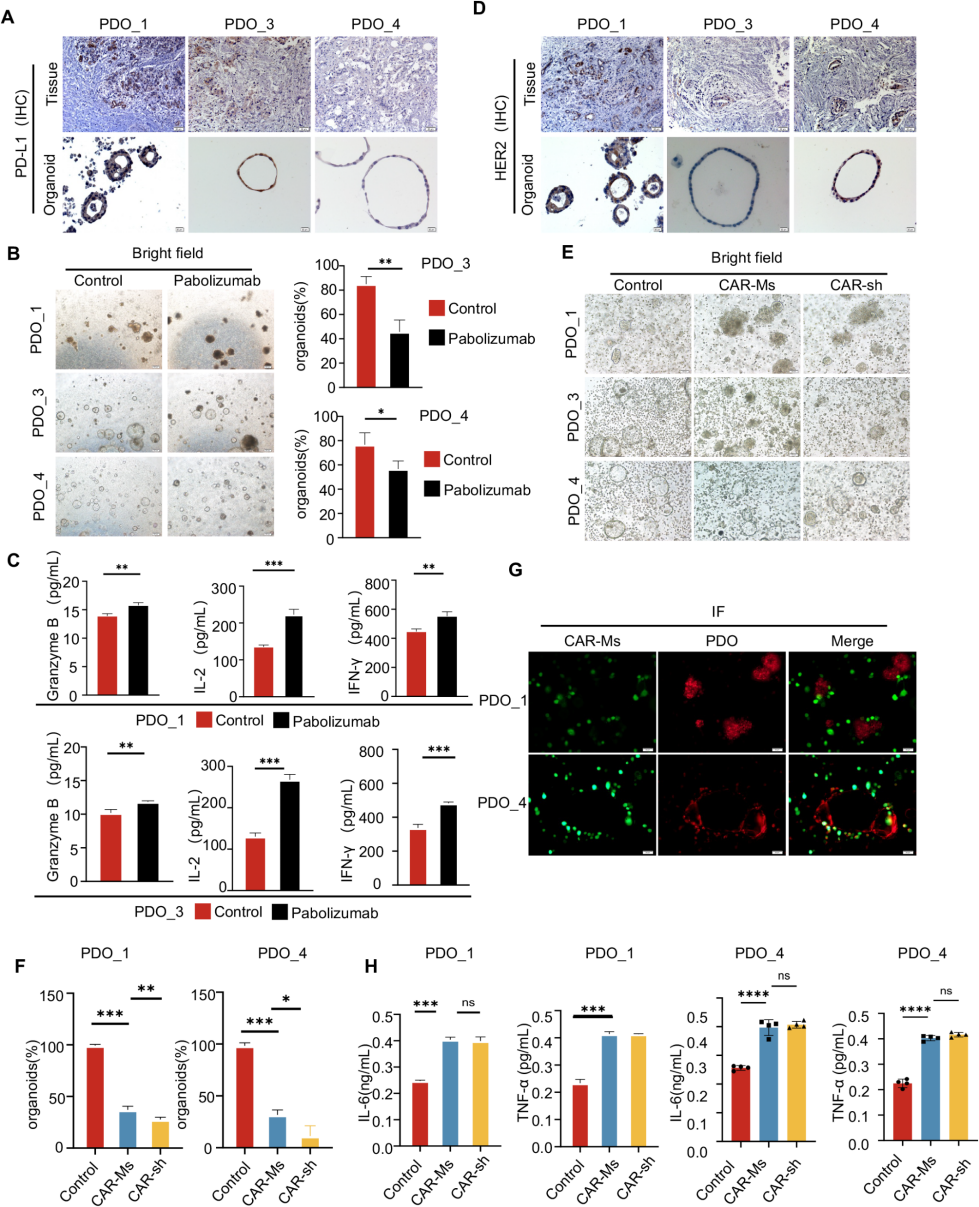
Assessing immunotherapy responsiveness to pembrolizumab and CAR-Ms therapies using PDOs. (**A**) Representative IHC images of paired PDAC tumors (scale bar: 50 μm), PDO (scale bar: 20 μm) for PD-L1. (**B**) Representative brightfield images of PDO_1, PDO_3, and PDO_4 co-cultured with PBMCs, subsequent to their exposure to either a control condition or Pembrolizumab treatment (scale bar: 200 μm). Additionally, it includes the results of organoid spheroid formation analysis. (**C**) Statistical analysis of the expression levels of granzyme B, IL-2, and IFN-γ was performed following co-culture PDO_1 or PDO_3 with PBMCs. (**D**) Representative IHC images of paired PDAC tumors (scale bar: 50 μm), PDO (scale bar: 20 μm) for HER2. (**E**) Representative brightfield images of PDO_1, PDO_3, and PDO_4 co-cultured with CAR-Ms or CAR-sh (scale bar: 200 μm). (**F**) Immunofluorescence analysis results for PDO_1, and PDO_4 following co-culture with CAR-Ms. (**G**) The immunofluorescence images show that CAR-Ms can specifically target HER2-positive cancer cells (PDO_1, 4) and enhance antitumor effectiveness. (**H**) Statistical analysis of the expression levels of IL-6 and TNF-α was performed following co-culture with CAR-Ms or CAR-sh

Considering that PDO_3 is less sensitive to chemotherapy and targeted drugs, we constructed a co-culture model of organoids with PBMCs from autologous to test the effect of the PD-1 inhibitor pembrolizumab [32]. First, we detected the expression of PD-L1 in PDO_1, PDO_3, and PDO_4. Before conducting further experiments, we analyzed the HLA typing results of PDO_1, PDO_3 and PDO_4 to confirm their origin. This analysis provided crucial information for the construction of the co-culture model and the subsequent interpretation of the experimental results (Sup Fig. 8). For the co-culture experiment, we chose the organoid culture medium as the optimal medium. Although T-cell culture medium is beneficial for the growth, activation, and functional performance of T-cells, our experiment focuses on the cytotoxicity of immune cells against tumor cells. The organoid culture medium can maintain the structural and functional integrity of organoids, mimic the in-vivo environment to promote interactions between cells, enhance the effectiveness of the experiment, reduce irrelevant interference, improve data reliability, and also reduce non-specific killing, helping to more accurately evaluate immune - mediated cytotoxicity. To verify the rationality of this choice, we carried out additional experiments and provided supplementary data. T-cells were cultured in T-cell culture medium and organoid culture medium for 72 h, respectively. Subsequently, cell viability assays and quantitative polymerase chain reaction (q-PCR) assays were performed to evaluate the viability and functional status of the T-cells. The experimental results showed that there were no significant differences in the viability and functional indices of T-cells under the two culture medium conditions. This finding indicates that the use of organoid culture medium does not affect the viability and functions of T-cells within a 72-hour culture period (Sup Fig. 9). The results showed that the expression of PD-L1 both in the original tumor and PDO_1/3 was positive, while PDO_4 was negative (Fig. 6A). Subsequently, we used the PDO and PBMC co-culture model to evaluate the effect of pembrolizumab (an ICIs). The results showed that in PDO_1 and PDO_3 with positive PD-L1 expression, the organoids lost their original 3D structure, turned black, and there was a significant decrease in both the diameter and spheroid formation rate (Fig. 6B, Sup Fig. 11A). These findings suggest that pembrolizumab may trigger the destruction and demise of PD-L1-positive organoids by stimulating PBMC-mediated immune responses. We then collected the supernatant from the co-culture system and quantified the levels of IL-2, granzyme B, and interferon-γ. The results showed that the release of cytokines in the pembrolizumab group was markedly higher than that the control group (Fig. 6C). However, in PDO_4, the cytokine levels did not differ significantly from those in the control group (Sup Fig. 11B). These results indicate that under 3D co-culture conditions, immune cells were effectively activated, demonstrating that this system successfully mimics the effects of ICIs in vitro. Furthermore, PD-L1-positive organoids displayed significant tumor-killing effects following pembrolizumab treatment, further validating the reliability of this co-culture system in assessing the efficacy of ICIs.

Adoptive cell therapy is a crucial part of immunotherapy, but its effectiveness against solid tumors is limited [33]. To address this, we developed a co-culture model utilizing PDO and CAR-macrophages (CAR-Ms) to more accurately replicate the tumor microenvironment and assess therapeutic outcomes. Initially, we engineered CAR-Ms by combining a humanized single-chain variable fragment with FcγRIIa and silencing SIRPα using shRNA. CAR-shSIRPα-M cells showed an M1-like phenotype and strong cytotoxicity against HER2-positive tumor cells [18]. Therefore, we identified high levels of HER2 expression in PDO_1 and PDO_4, which are suitable models for evaluating HER2-targeted therapies (Fig. 6D). Finally, in a co-culture system comprising PDOs and CAR-Ms, we observed that CAR-Ms had a significant tumoricidal effect on PDOs with elevated HER2 expression, diminished sphere-forming ability, and triggered tumor cell apoptosis (Fig. 6E-F). This suggests that CAR-Ms can inhibit tumor cell proliferation and growth, thereby validating their cytotoxic potential in HER2-positive tumors.

Moreover, immunofluorescence technology was applied to analyze co-culture systems. CAR-Ms were observed in close proximity to PDOs (PDO_1 and PDO_4), infiltrating the tumor microenvironment and engaging directly with tumor cells, which underscores their capacity to specifically target HER2-positive cancer cells (Fig. 6G). Additionally, we noted enhanced secretion of TNF-α and IL-6, indicative of a robust immune response (Fig. 6H, Sup Fig. 3B). CAR-Ms enhance antitumor effectiveness by modulating the inflammatory response within the tumor microenvironment. These results suggests that the co-culture model of PDO and CAR-Ms more accurately simulates the tumor microenvironment and serves as a valuable tool for evaluating the efficacy of immunotherapies.

## Discussion

The main characteristics of PDAC are poor prognosis and high mortality rate. In recent years, progress in sequencing technology, as well as the development of chemotherapeutic drugs, targeted drugs, and immunotherapeutic drugs, has made the treatment options for PDAC more diverse. However, it is difficult to summarize the genomic diversity and individual differences among patients based solely on their clinical status and staging, and there is an urgent need for an ideal preclinical model that can interpret patient heterogeneity [34]. The emergence of PDO models has provided a new method for solving this problem.

In this study, we established PDO and corresponding PDOX models derived from PDAC, integrating patient sequencing and clinical data to assess the efficacy of PDO models as preclinical tools and to explore their potential applications. Few preclinical models accurately reflect the clinical and molecular heterogeneity of patients, which limits the further development of treatment strategies. While the use of PDOs can address this issue, further research is needed to determine whether PDOs can retain the corresponding molecular characteristics of the patients over time and maintain similar drug responses in vitro. To maintain genetic stability, we selected PDOs within 5–10 passages for sequencing and subsequent experiments [35]. It is not only possible to obtain a sufficient number of tumor cells to support the need for high-throughput drug screening, but also to retain the main tumor-driving genes and drug targets of the original tumor tissue using whole exome sequencing. This 3D culture in vitro only led to mutations in approximately 3% of the genes, which is a significant advancement compared to traditional primary cell culture. In terms of time, throughput, and preservation of the mutational characteristics and genes of the original tumor tissue, PDOs essentially meet the requirements of a preclinical model.

To further validate the effectiveness of PDOs for high-throughput drug screening, we established corresponding PDOX models using xenograft PDOs in NCG mice. Four PDOX models were successfully obtained from five organoids that could be stably passaged. Both PDO_1 and matched PDOX displayed the highest sensitivity to gemcitabine, while their sensitivity to other drugs was markedly lower. Furthermore, the combination of gemcitabine and cisplatin yielded a greater tumor cell inhibition rate than either drug alone did. The drug sensitivity outcomes from PDO_1 were further validated in patient 1. The regimen combining of gemcitabine and cisplatin showed better tumor growth reduction in both PDO_1 and its matching PDOX model, also effectively suppressing tumor growth in the patient. These findings suggested that PDOs and matched PDOX models can function as viable preclinical models, parallel to clinical settings, in guiding the selection of therapeutic drugs for patients. The combination of in vivo and in vitro experimental results greatly enhances the credibility of PDOs in drug screening.

Our analysis, which includes sequencing, drug screening, PDOX in vivo experiments, and clinical patient data, reveals significant variability in the tumor-killing efficacy of different drugs and substantial differences among individual patients. This indicates that while the expression of tumor markers can predict the prognosis of treatment to an extent, the heterogeneity among patients significantly affects the accuracy of treatment prognosis predictions. By analyzing the sequencing results from various drug-sensitive groups and conducting expression and prognostic analyses of selected genes within TCGA, we identified UGT1A10 and CA9. These genes are highly expressed in patients with drug insensitivity, show significant differences in expression between tumor and normal tissues, and are associated with poorer patient survival rates.

The potency of anti-cancer drugs is not only due to their direct cytotoxicity to tumor cells, but also due to their capacity to facilitate drug transport and delivery to precise molecular targets [36] UGT1A10, a member of the UDP-glucuronosyltransferase (UGT) enzyme family, plays a crucial role in drug metabolism. This enzyme is integral to drug inactivation and excretion, potentially underpinning drug resistance in patient. UGT1A10’s primary function is to transfer glucuronic acid to various endogenous and exogenous compounds, thereby enhancing their water solubility and promoting their elimination from the body. This process could significantly influence the ultimate efficacy of drug therapy [37, 38]. Our findings suggest that variations in UGT1A10 expression may be correlated with differences in drug sensitivity among PDOs. Preliminary analysis of UGT1A10 expression levels in TCGA database revealed higher expression in pancreatic cancer patients, which was associated with a poorer prognosis. In our study, knockdown of the UGT1A10 gene in PDO_3 induce better tumor-killing effects for gemcitabine, paclitaxel, cisplatin, irinotecan, indicating that the expression of UGT1A10 affected drug sensitivity. Furthermore, validation experiments demonstrate that high levels of UGT1A10 expression are detected in the gemcitabine-resistant PDAC cell line, suggesting that UGT1A10 may be a potential target for drug resistance. All these results indicate that genetic diversity should be considered in personalized cancer treatment research before concentrating on tumor markers expression, to advance personalized treatment strategies based on individual patient differences.

The emergence and development of immunotherapy has provided new ideas for the treatment of PDAC, especially for pancreatic patients that do not respond well to chemotherapy. Although PDOs capture patient heterogeneity to a certain extent, they lack a tumor microenvironment (TME) and are difficult to use for evaluating immunotherapy [8, 39]. Establishing a co-culture model of PDOs and PBMCs may offer a promising avenue for assessment of immunotherapies. While the expression of UGT1A10 influences the drug sensitivity of PDO_3 to commonly used clinical medications, the presence of PD-L1 in both the original tumor and PDO_3 was consistently positive. This indicates the potential for selecting PDO_3 as a candidate for ICIs immunotherapy. In the assessment of the effect of the immunotherapy drug pembrolizumab, we found that PDO_3 in the co-culture model exhibited enhanced tumor-killing effects and elevated cytokine release.

Macrophages exhibit a strong capacity for tumor infiltration and constitute the predominant immune cell population in solid tumors. The potential of macrophage-mediated phagocytosis as a cancer treatment is promising [40]. To validate the targeted killing capability of CAR-modified macrophages, we co-cultured CAR-Ms exhibiting an M1-like phenotype and potent cytotoxicity against HER2-positive tumor cells with PDO expressing high levels of HER2. These CAR-Ms were found to suppress tumor cell proliferation and growth. CAR-Ms significantly enhance their antitumor potency by modulating the inflammatory response within the TME. This co-culture model provides a more precise simulation of the TME. These experimental outcomes confirmed that immune cells can survive and perform effectively within the PDO co-culture system, underscoring the potential of this model for assessing the efficacy of immunotherapies. Future studies could enhance this model by integrating additional immune cell types and elements of the tumor microenvironment, thus creating a more holistic representation of the intricate in vivo tumor ecosystem. Such improvements would facilitate more nuanced evaluations of patient-specific tumor-immune dynamics, laying the groundwork for the development of personalized immunotherapies for patient refractory to chemotherapy. By refining this model, we provide more precise instruments for the advancement of precision medicine, ultimately enhancing treatment outcomes for cancer patients.

## Conclusion

PDAC treatment requires personalized strategies that consider genomic diversity beyond traditional tumor markers. In this regard, we have successfully established PDOs and their corresponding PDOX models. PDOs maintain the mutation landscape of the original tissue, with differential genes causing individual variation among PDOs. To some extent, PDO and matched PDOX can serve as parallel preclinical models to predict clinical responses. The two PDOs exhibited significantly different drug sensitivities owing to the number of differential genes, particularly the variation in UGT1A10 expression. Further use of a co-culture system of PDO and immune cells can assess the efficacy of immunotherapy in vitro. Thus, combining the molecular characteristics and therapeutic analysis of PDOs can predict clinical responses and guide personalized PDAC treatment.

## Electronic supplementary material

Below is the link to the electronic supplementary material.

Supplementary Material 1

## Data Availability

No datasets were generated or analysed during the current study.

## Abbreviations

PDAC: Pancreatic ductal adenocarcinoma
PDOX: Patient-derived organoid xenograft
PBMC: PDO-peripheral blood mononuclear cells
CAR-Ms: Engineered chimeric antigen receptor macrophages
RNA-seq: RNA-sequencing
SNV: Single nucleotide variants
InDel: Insertions and deletions
AUC: The area under the drug-dose curve
TME: Tumor microenvironment
