# Supplementary material for: Combining molecular characteristics and therapeutic analysis of PDOs predict clinical responses and guide PDAC personalized treatment
Source: J Exp Clin Cancer Res. 2025 Feb 26;44:72. doi: 10.1186/s13046-025-03332-8 (PMC11863571; doi:10.1186/s13046-025-03332-8)
Supplement: Supplementary file 1 — Supplementary Material 1 [file 13046_2025_3332_MOESM1_ESM.docx]

**Supplementary**


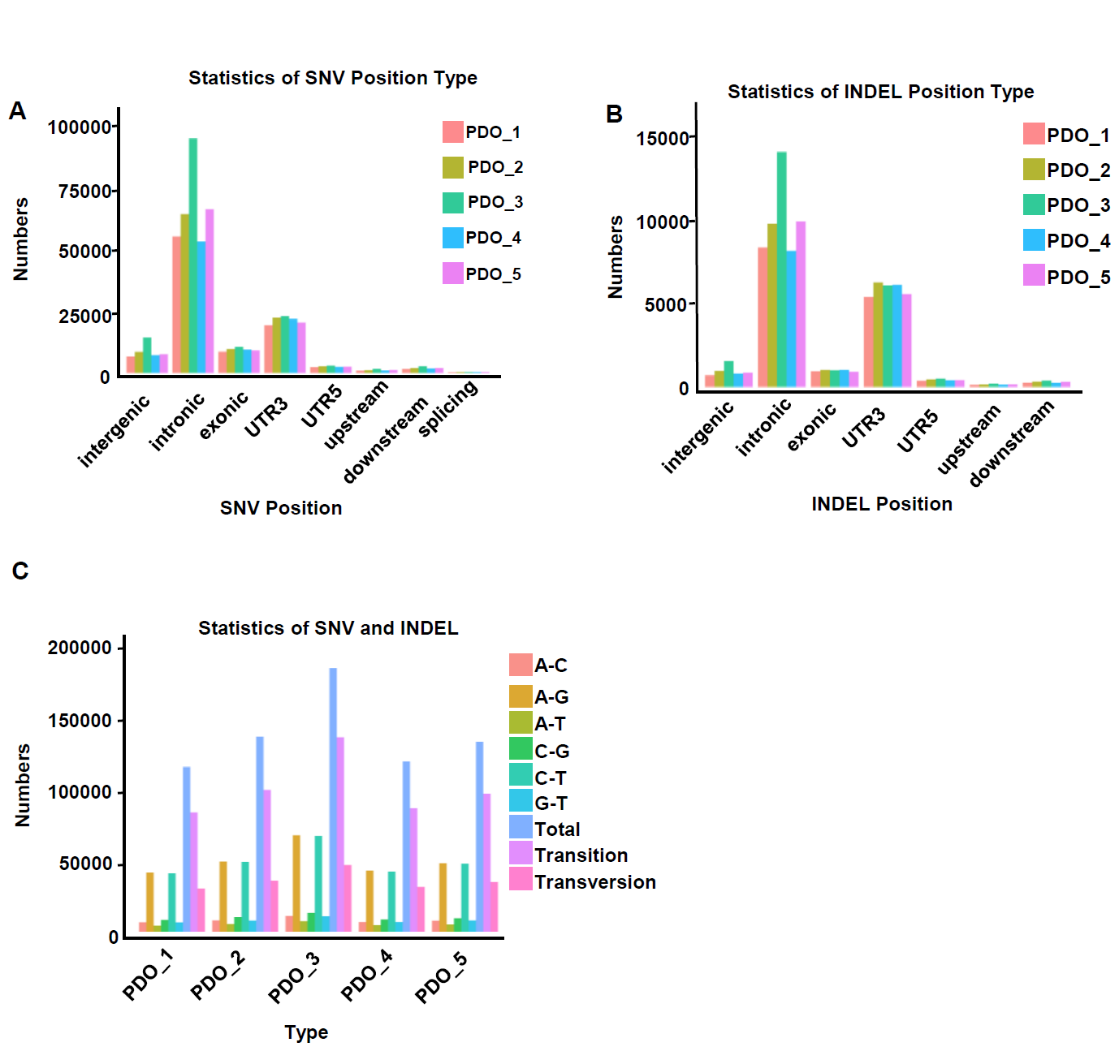


**Figure S1: The results of SNV and InDel among different PDOs**

A. The statistics of SNV position type mutations in 5 PDOs were respectively represented. B. The statistics of INDEL position type mutations in 5 PDOs were respectively represented. C. The statistics of INDEL and SNV base substitutions mutations in 5 PDOS.


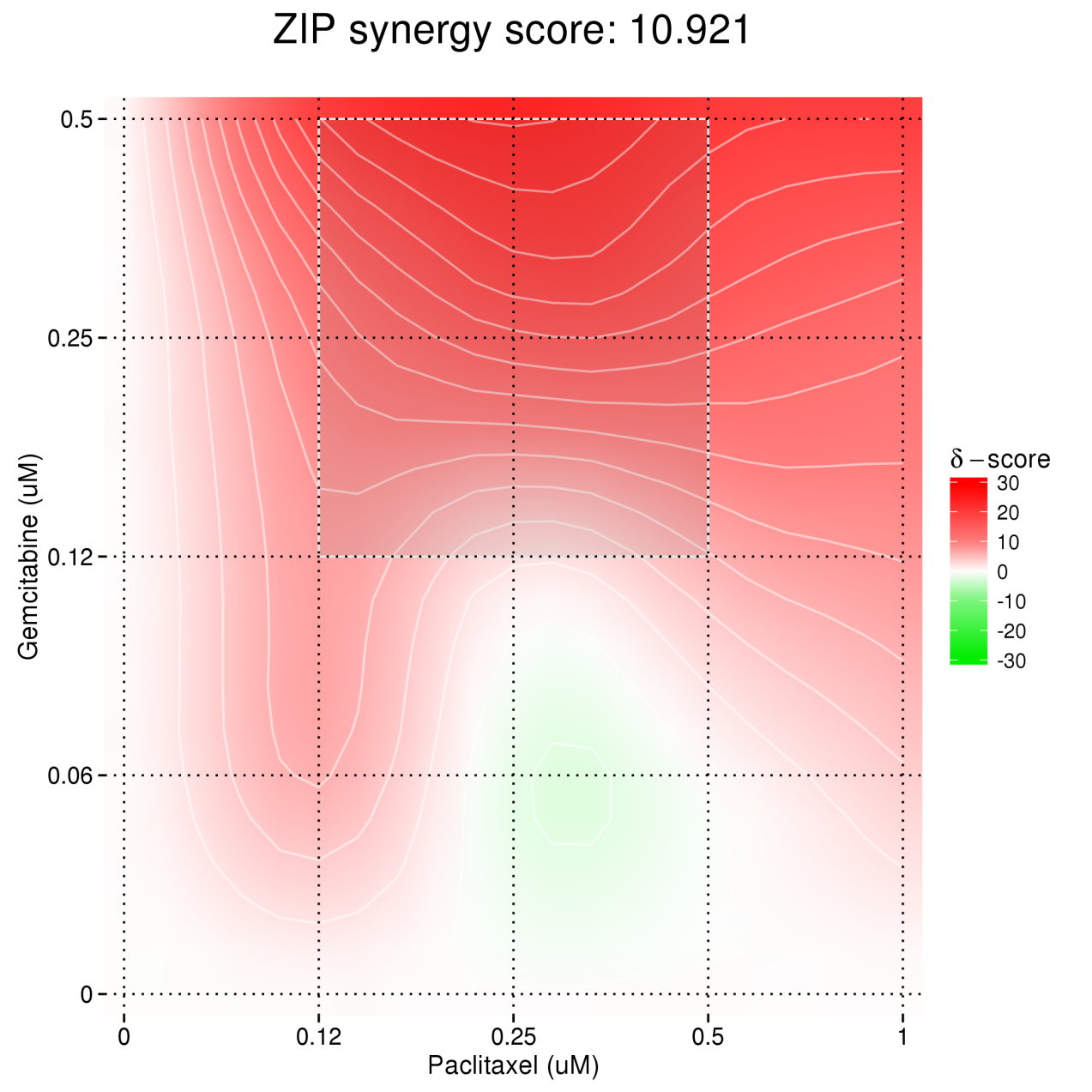


**Figure S2: Synergy score of Gemcitabine and Paclitaxel analyzed using Synergyfinder in PDO_1**


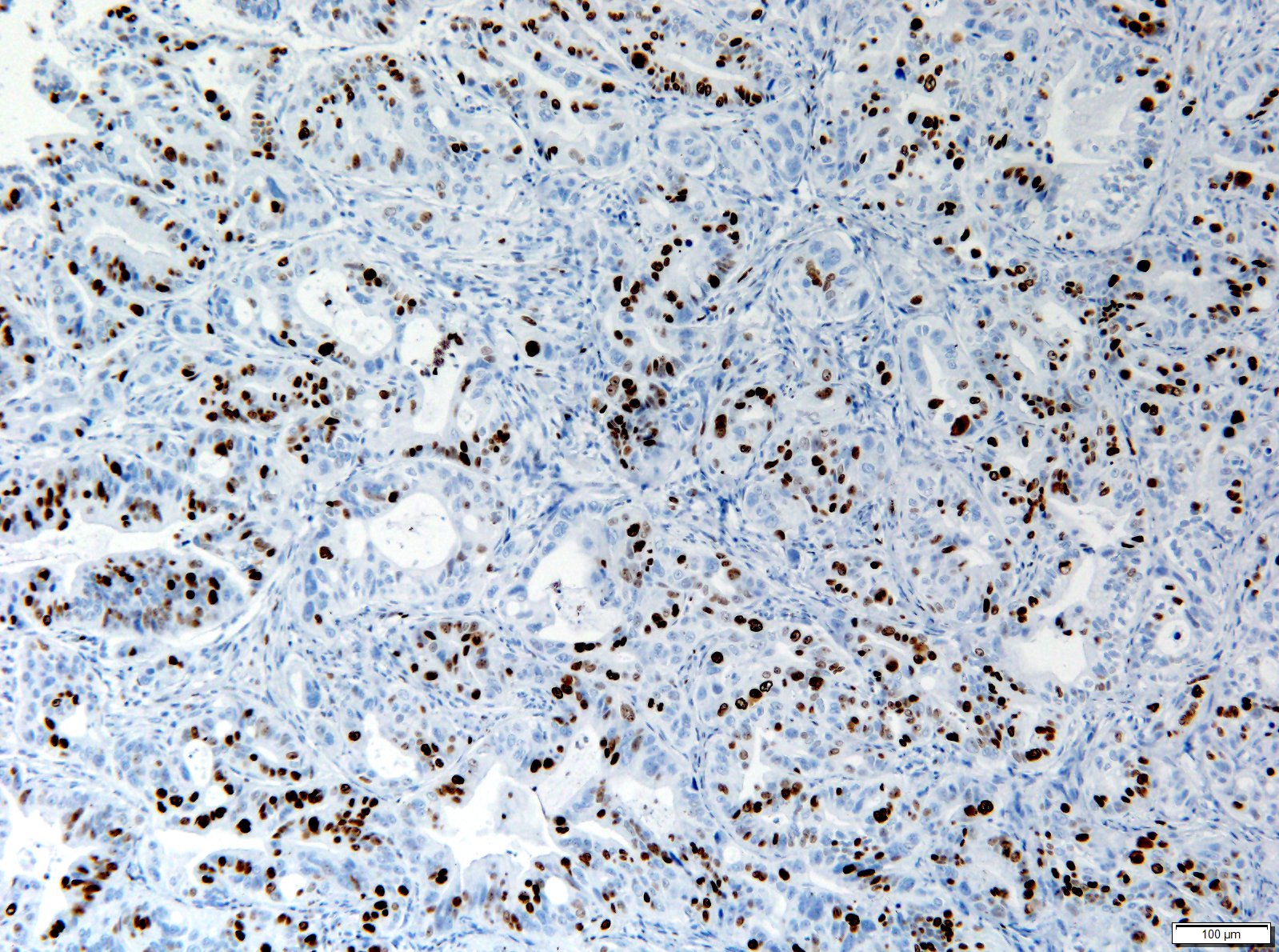

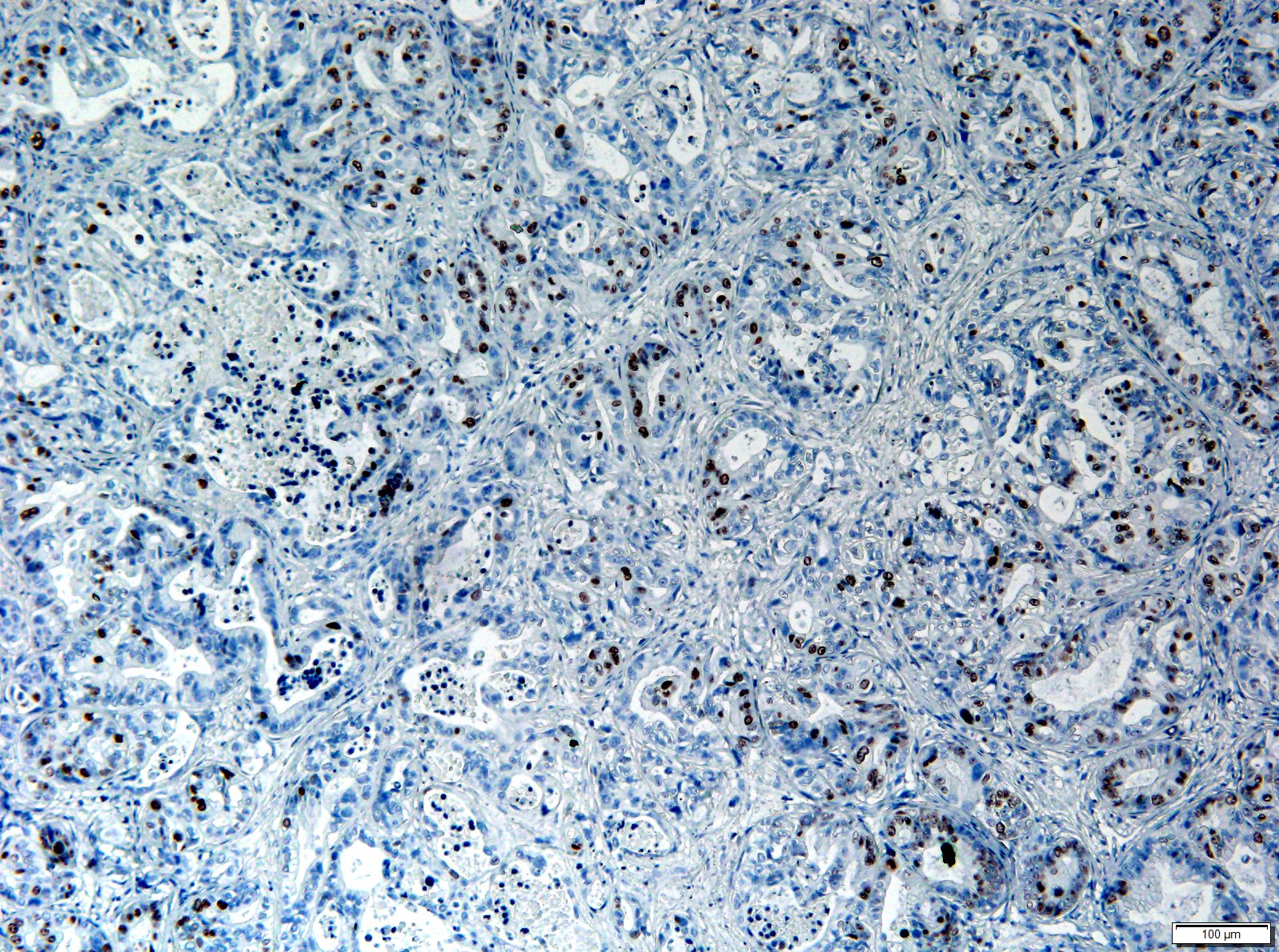

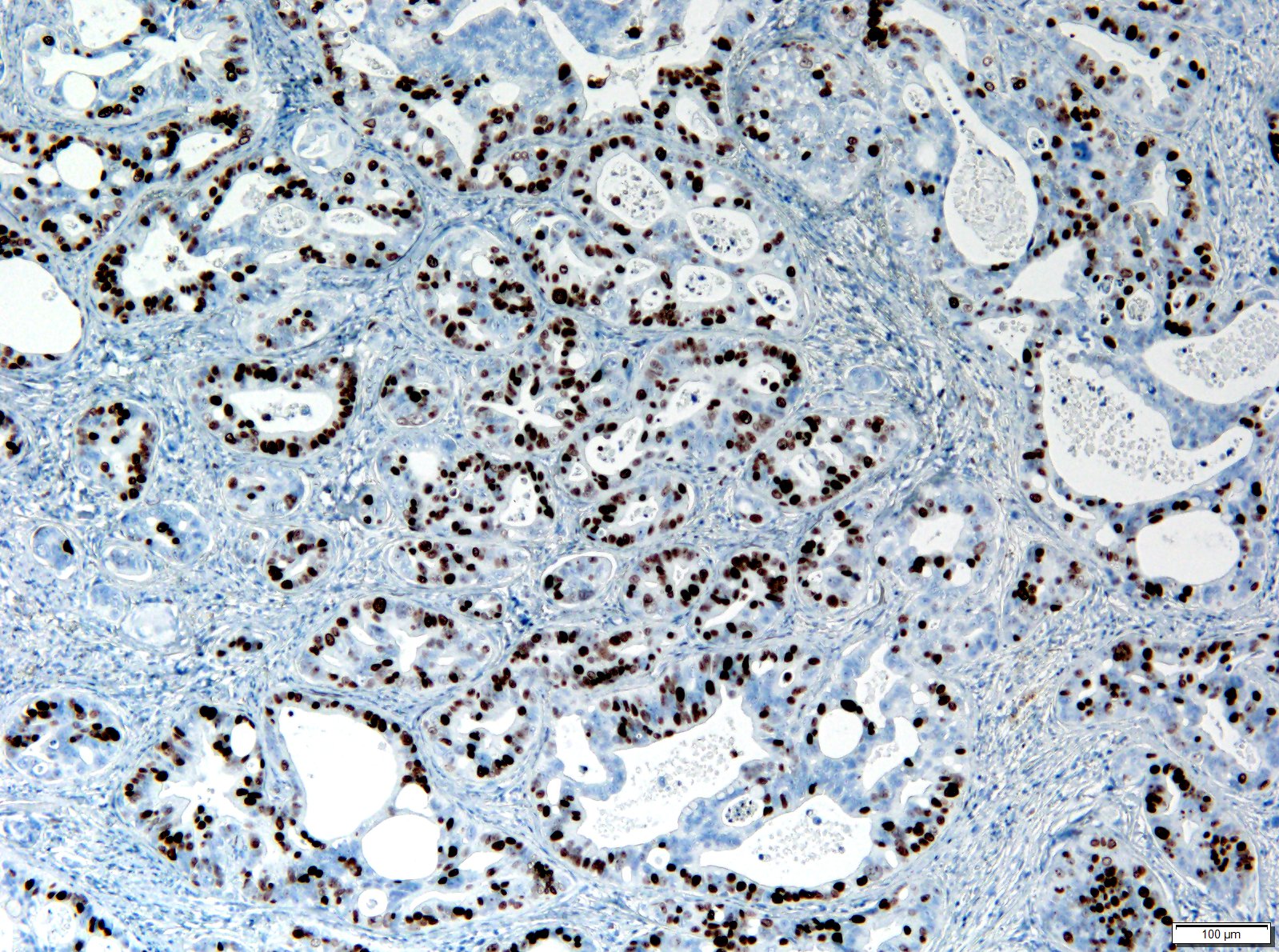


PDOX Ki-67(IHC)

Control

Combination

Gemcitabine

**Figure S3: The expression of Ki-67 in PDOX models across different treatment groups: control, gemcitabine-treated, and combination-treated.**


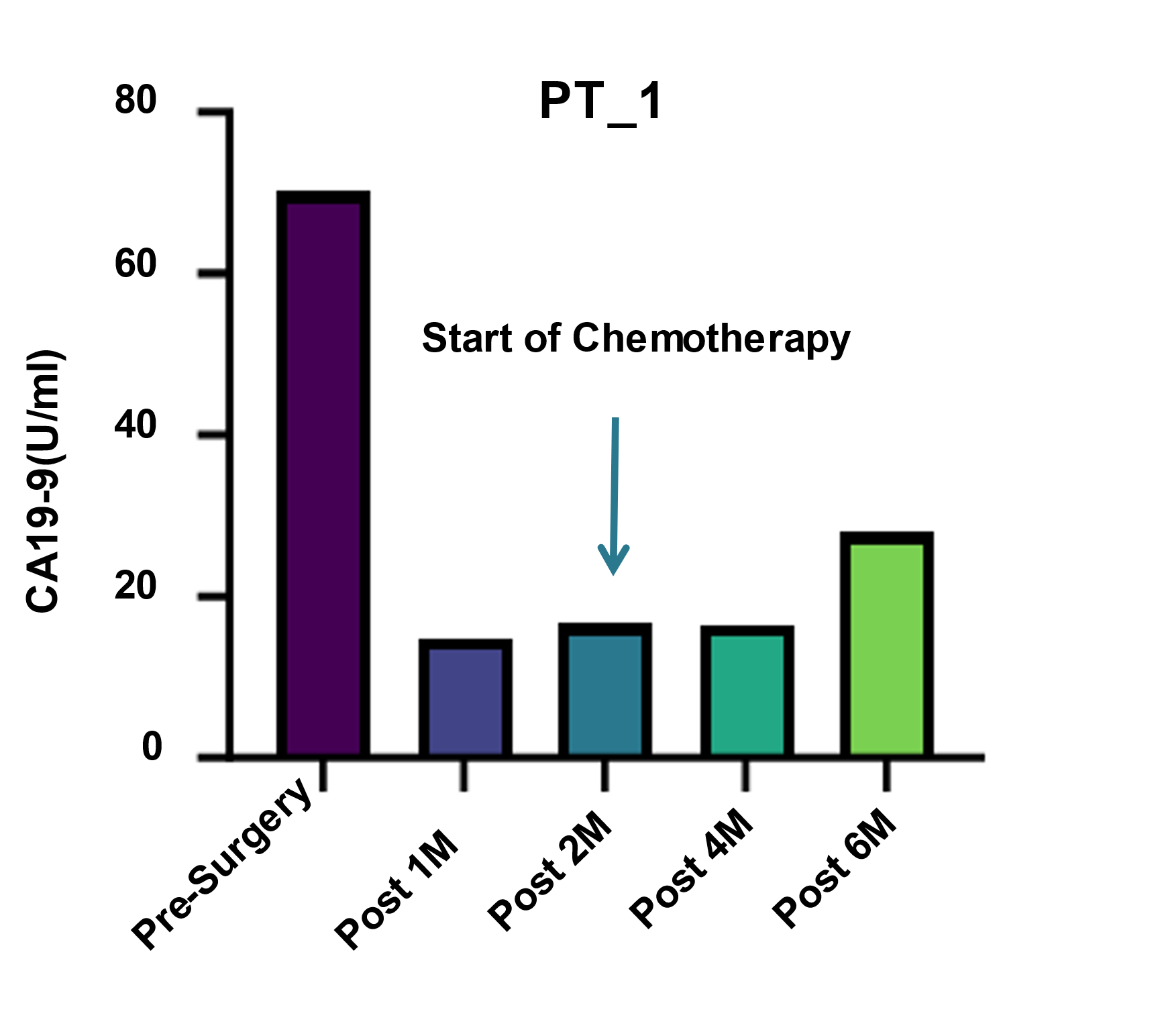


**Figure S4: The combination therapy of gemcitabine and paclitaxel effectively reduced the CA19-9 levels in serum of patient PT_1. M means month.**


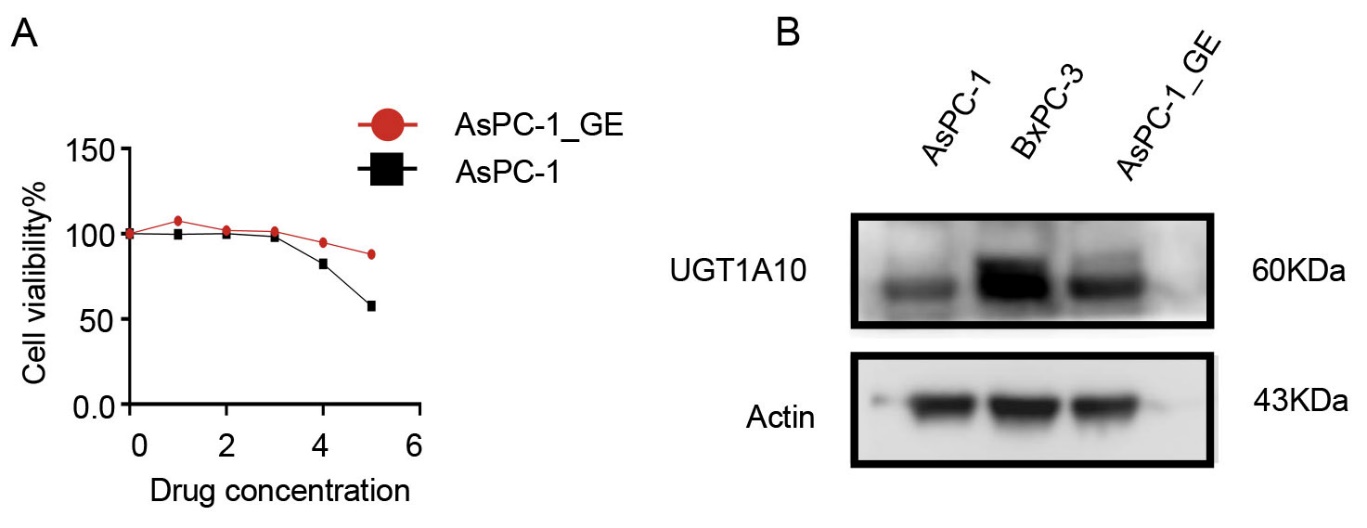


**Figure S5:** **Expression of UGT1A10 is upregulated in gemcitabine-resistant PDAC cell lines**

A. PDAC cell line AsPC-1 develops resistance following induction by Gemcitabine.

B. The Gemcitabine-resistant cell line AsPC-1_GE exhibits increased UGT1A10 expression compared to the parental AsPC-1 line. Additionally, the PDAC cell line BxPC-3 shows the high level UGT1A10 expression.


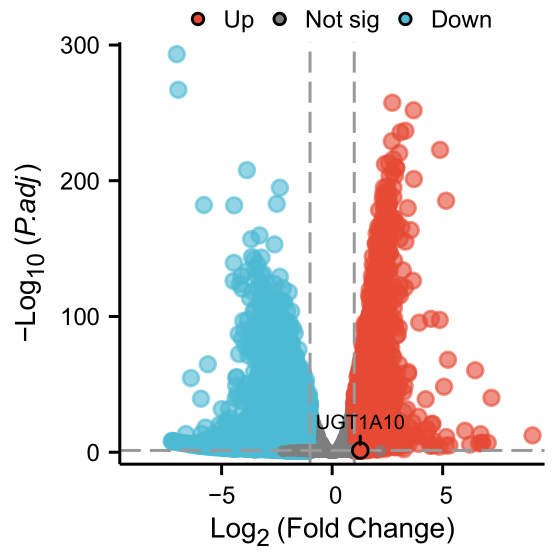


**Figure S6:** **The volcano plot compares bulk RNA-seq data between the human PDAC cell line CFPAC-1 and its gemcitabine-resistant cells.**

Red points denote upregulated genes, blue points represent downregulated genes, and gray points signify genes without significant differences. The encircled point highlights the upregulated differential gene UGT1A10.


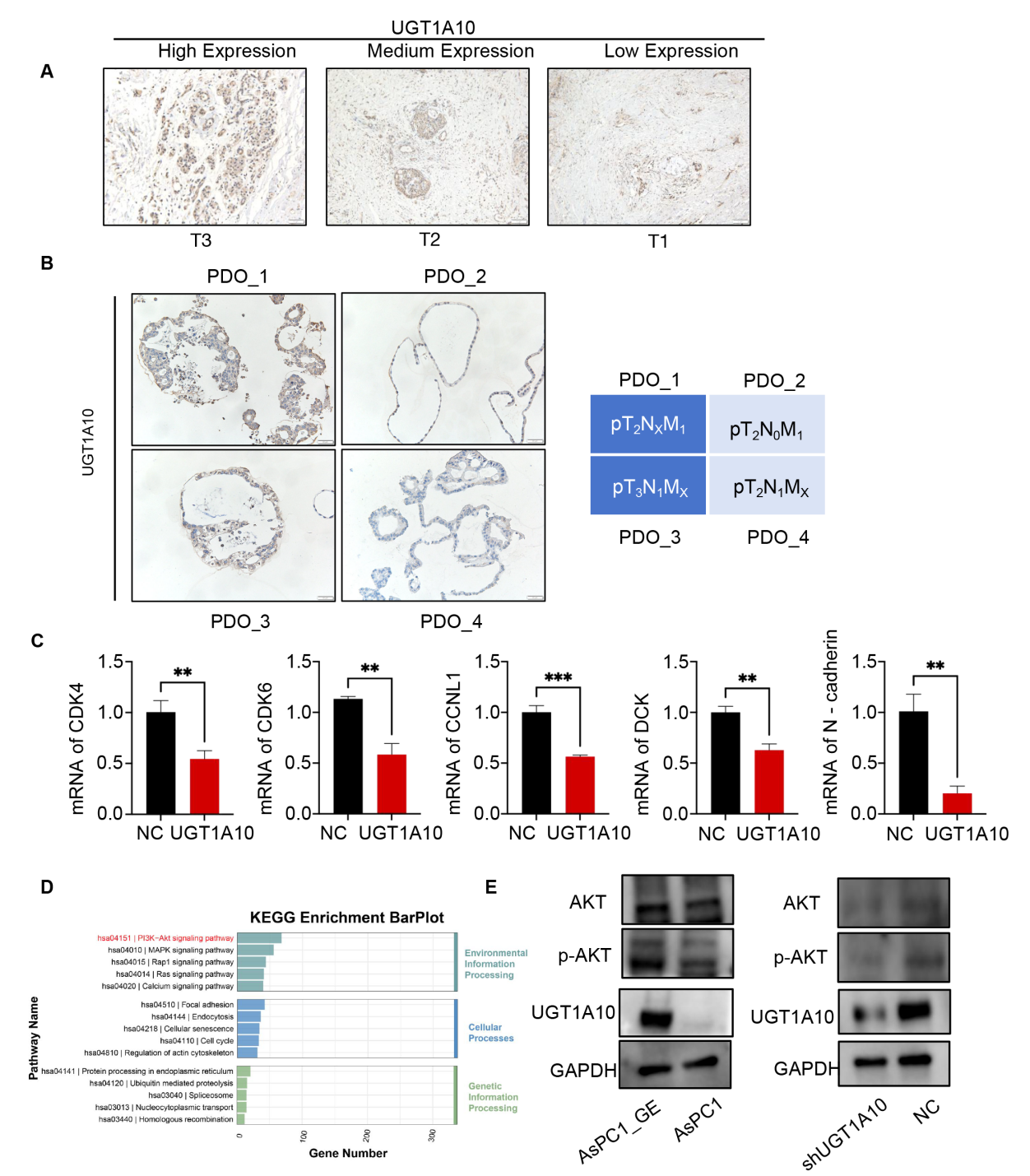


**Figure S7: Research on the role of UGT1A10 in pancreatic cancer pathological grade and drug resistance mechanism.**

A. Upon conducting immunohistochemical analysis of clinical pancreatic cancer specimens, we observed that the expression level of UGT1A10 is positively correlated with the pathological grade of pancreatic cancer (n=9). B. In histological analyses of patient-derived organoids (PDOs), the expression level of UGT1A10 was found to exhibit a positive correlation trend with the pathological grade of the corresponding clinical pancreatic cancer. C. After knocking down UGT1A10 in the organoid PDO_3, the related drug-resistant genes (including CDK4, CDK6, CCNL1, DCK, N-cadherin.) also showed a downward trend as detected by q-PCR (Sup Table 5). D. The KEGG enrichment bar - chart shows that UGT1A10 is involved in the regulation of the drug-resistance mechanism through the PI3K-akt pathway. E. Compared with the parental AsPC-1 cell line, the Gemcitabine-resistant cell line AsPC-1_GE shows increased expression of UGT1A10, p-AKT, and AKT. Compared with the NC group, the organoids with UGT1A10 knockdown exhibit decreased expression of p-AKT and AKT.


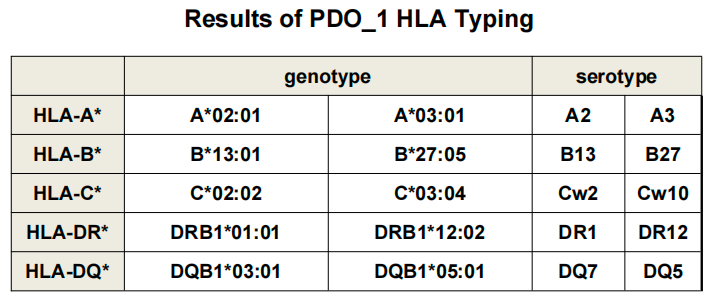

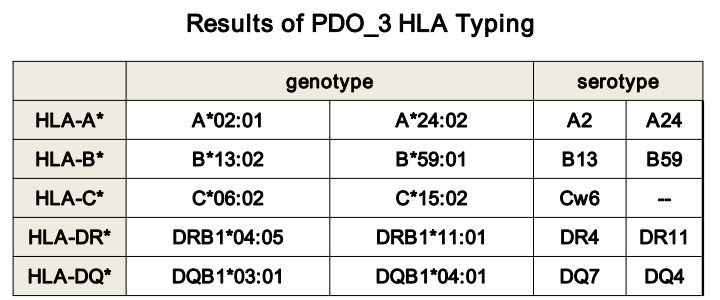

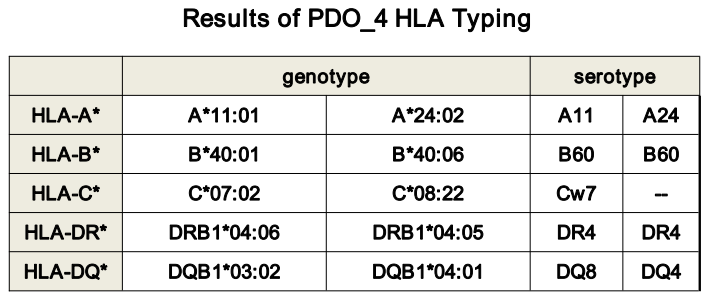


**Figure S8: The HLA typing results of PDO_1, PDO_3 and PDO_4.**


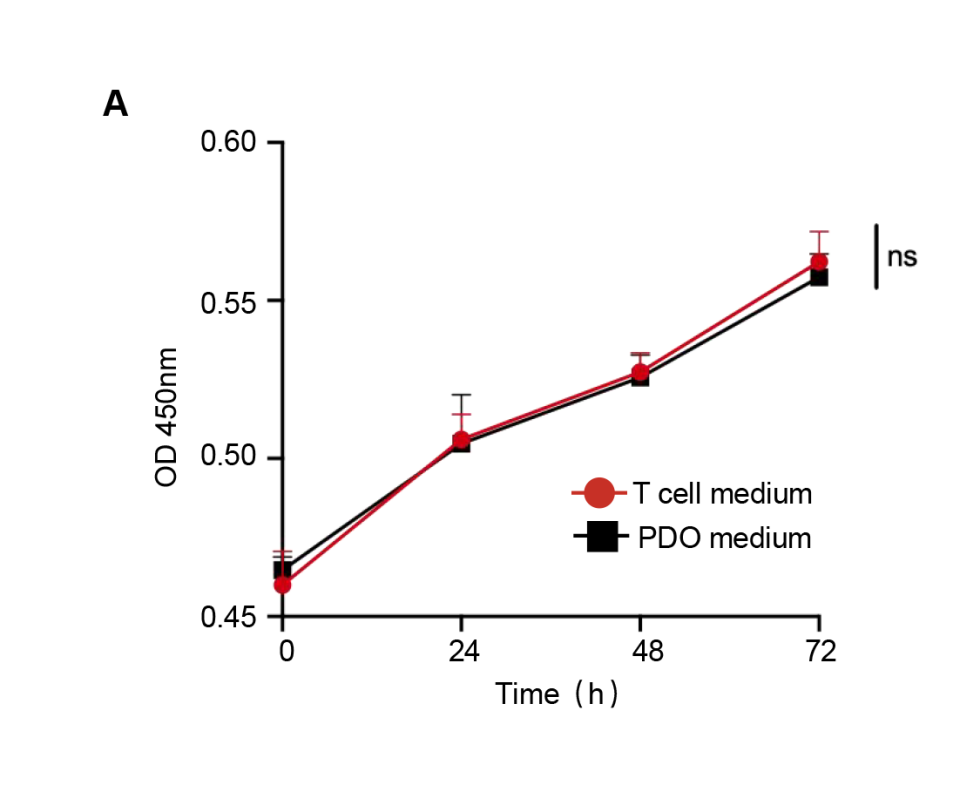


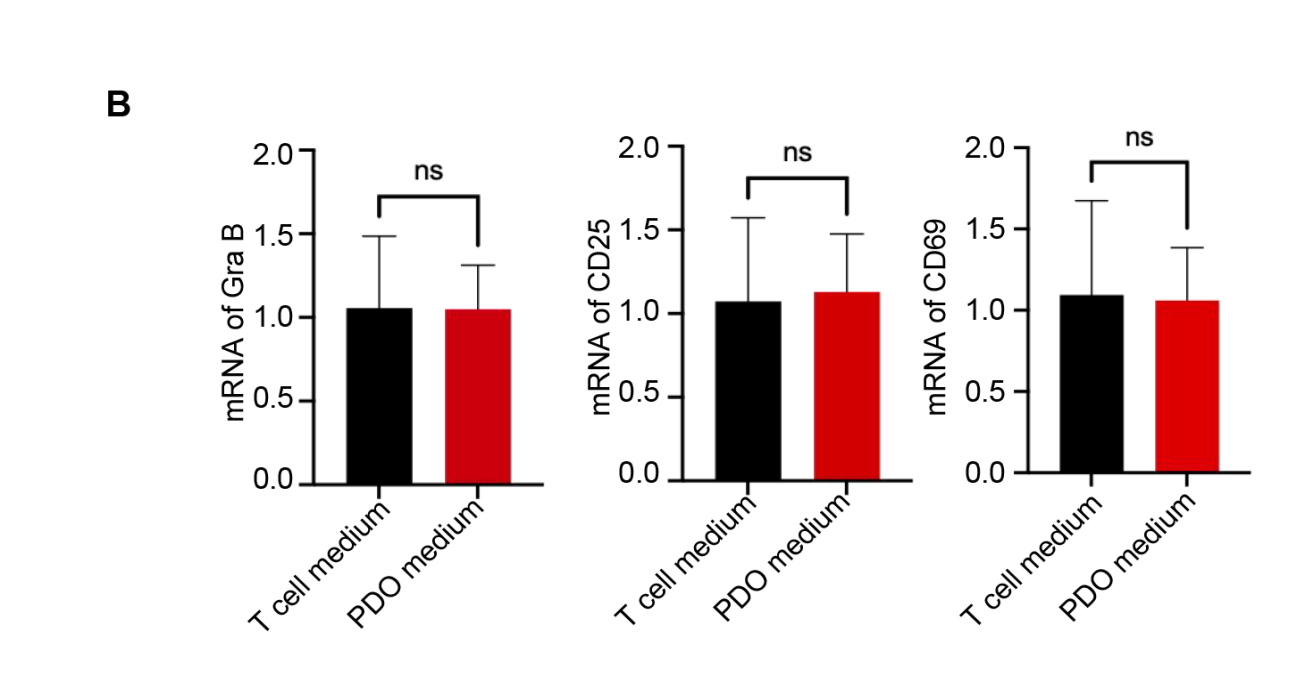


**Figure S9: Comparative analysis of T cell viability and functionality in T cell medium versus PDO medium"**

A: The proliferation of T cells in T cell medium (red) and PDO medium (black). The viability and activity of T cells were quantitatively assessed at 0, 24, 48, and 72 hours using the optical density (OD) at 450 nm as an indicator. B: The mRNA expression levels of Granzyme B (Grzm B), CD25, and CD69 (Sup Table 5) in T cells cultured in T cell medium and PDO medium were evaluated.


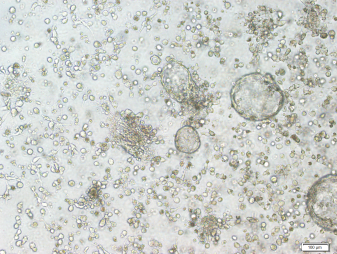

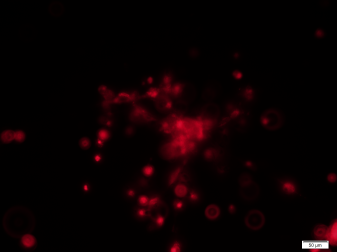

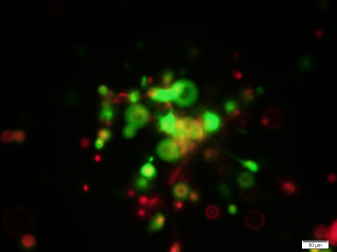

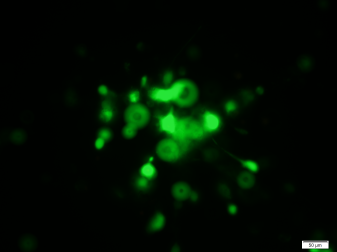


Bright field

Merge

IF

PDO

CAR-Ms

**Figure S10: Immunofluorescence (IF) images demonstrate the interaction between CAR-Ms (in red) and PDOs (in green). From left to right are the bright field image, the fluorescence - labeled image of CAR-Ms, the fluorescence labeled image of PDOs, and the merged (Merge) image of those two.**

**
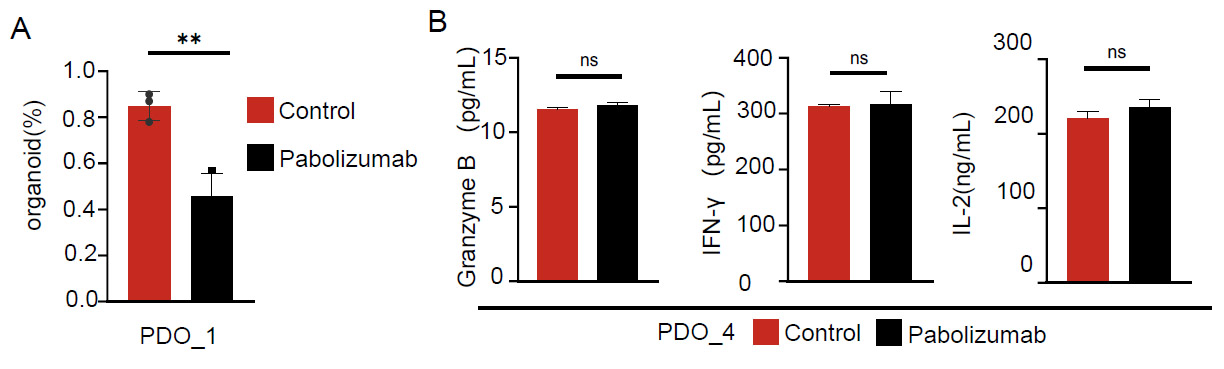
**

**Figure S11:** **Evaluating the immunotherapy sensitivity of pabolizumab and CAR-Ms through PDOs.**

A. Results of organoid spheroid formation analysis of PDO_1 co-cultured with PBMCs. B. Statistical analysis of the level of IL-2, granzyme B, and IFN-γ was conducted after co-culturing with PBMCs in PDO_4.

**Table S1. Information on pancreatic cancer patients**

| **Serial No** | **ID** | **Gender** | **Age** | **Differentiation** | **Case Subtype** | **Sample Site** | **Tumor Stage** |
| --- | --- | --- | --- | --- | --- | --- | --- |
| 1 | PT_1 | Male | 52 | Medium | PDAC | Pancreatic Head | T2NxM1 |
| 2 | PT_2 | Female | 60 | Medium | PDAC | Pancreatic Head | T2N0M1 |
| 3 | PT_3 | Male | 69 | Medium | PDAC | Pancreatic Head | T3N1Mx |
| 4 | PT_4 | Male | 45 | Medium | PDAC | Pancreatic Head | T2N1Mx |
| 5 | PT_5 | Male | 72 | Medium/High | PDAC | Pancreatic Head | T2N1Mx |

**Table S2. Drug concentrations of five first-line therapies for pancreatic cancer**

| Drug Concentration  (ng/mL) | 5 | 4 | 3 | 2 | 1 |
| --- | --- | --- | --- | --- | --- |
| Gemcitabine | 7900 | 1580 | 316 | 63.2 | 12.64 |
| 5-Fluorouracil | 1300 | 260 | 52 | 10.4 | 2.08 |
| Cisplatin | 850 | 170 | 34 | 6.8 | 1.36 |
| Irinotecan | 2000 | 400 | 80 | 16 | 3.2 |
| Irinotecan | 3700 | 740 | 148 | 29.6 | 5.92 |

**Table S3.** **Information of 111drugs for treatment of PDAC**

| **Serial No** | **Drug** | **Serial No** | **Drug** |
| --- | --- | --- | --- |
| 1 | Axitinib (AG 013736) | 54 | Resminostat |
| 2 | Bortezomib (PS-341) | 55 | Tivantinib (ARQ 197) |
| 3 | Dasatinib (BMS-354825) | 56 | Evofosfamide (TH-302) |
| 4 | Gefitinib (ZD1839) | 57 | Devimistat (CPI-613) |
| 5 | Imatinib (STI571) Mesylate | 58 | Tacedinaline (CI994) |
| 6 | Lenalidomide (CC-5013) | 59 | Icotinib (BPI-2009H) |
| 7 | Nilotinib (AMN-107) | 60 | Daunorubicin (RP 13057) HCl |
| 8 | Sorafenib (BAY 43-9006) tosylate | 61 | Carmustine |
| 9 | Sunitinib (SU11248) malate | 62 | Isoquercitrin |
| 10 | Olaparib (AZD2281) | 63 | (-)-Arctigenin |
| 11 | Masitinib (AB1010) | 65 | Vinorelbine ditartrate (KW-2307) |
| 12 | Crizotinib (PF-02341066) | 66 | 6-Mercaptopurine (6-MP) Monohydrate |
| 13 | Vismodegib (GDC-0449) | 67 | Vinblastine (NSC-49842) sulfate |
| 14 | BI 2536 | 68 | 2-Deoxy-D-glucose (2-DG) |
| 15 | Everolimus (RAD001) | 69 | Ciprofloxacin hydrochloride hydrate |
| 16 | Docetaxel (RP56976) | 70 | Nimustine Hydrochloride |
| 17 | Paclitaxel (NSC 125973) | 71 | Chloropyramine hydrochloride |
| 18 | Capecitabine (RO 09-1978) | 72 | Abemaciclib (LY2835219) |
| 19 | Regorafenib (BAY 73-4506) | 75 | Osimertinib (AZD9291) |
| 20 | Thalidomide (K17) | 76 | PND-1186 (VS-4718) |
| 21 | Doxorubicin (Adriamycin) HCl | 77 | Salirasib |
| 22 | Fluorouracil (5-Fluorouracil | 78 | Dovitinib(TKI258)Lactate monohydrate |
| 23 | Methotrexate (CL-14377) | 79 | Sunitinib (SU11248) |
| 24 | Dacarbazine (NSC-45388) | 80 | Dasatinib Monohydrate |
| 25 | Epirubicin (IMI 28) HCl | 81 | Erlotinib (OSI-774) |
| 26 | Etoposide (VP-16) | 82 | PX-12 |
| 27 | Topotecan (NSC609699) HCl | 83 | Napabucasin (BBI608) |
| 28 | Vincristine (NSC-67574) sulfate | 84 | Nirogacestat (PF-03084014) |
| 29 | Carmofur | 85 | Riviciclib hydrochloride (P276-00) |
| 30 | Floxuridine (NSC 27640) | 86 | Samotolisib (LY3023414) |
| 31 | Tegafur (FT-207) | 87 | GSK2256098 |
| 32 | Ifosfamide (NSC109724) | 88 | Tucidinostat (Chidamide) |
| 33 | Mercaptopurine (6-MP) | 89 | Harringtonine |
| 35 | Ruxolitinib (INCB018424) | 90 | Talabostat (PT-100) |
| 36 | Pirarubicin (NSC-333054) | 91 | Acelarin (NUC-1031) |
| 37 | Tipifarnib (R115777) | 92 | Irinotecan (CPT-11) |
| 38 | Pimasertib (AS-703026) | 93 | Fluzoparib (SHR-3162) |
| 39 | Gemcitabine (LY-188011) | 94 | Metarrestin (ML246) |
| 40 | Thioguanine (NSC 752) | 95 | Trametinib DMSO solvate |
| 41 | Teniposide | 96 | Irinotecan hydrochloride |
| 42 | Lomustine | 97 | Rucaparib Camsylate |
| 43 | Hydroxyurea (NSC-32065) | 98 | Pimonidazole |
| 44 | Gimeracil | 100 | Oleandrin (PBI-05204) |
| 45 | Cyclophosphamide (NSC-26271) Monohydrate | 101 | Cisplatin (NSC 119875) |
| 46 | Irinotecan (CPT-11) HCl Trihydrate | 102 | Exatecan Mesylate |
| 47 | Momelotinib (CYT387) | 103 | Endovion (NS 3728) |
| 48 | (S)-10-Hydroxycamptothecin | 104 | Relacorilant |
| 49 | Mitoxantrone (NSC-301739) 2HCl | 105 | Gemcitabine (LY-188011) HCl |
| 50 | Rabusertib (LY2603618) | 106 | Carboplatin (NSC 241240) |
| 51 | Rabusertib (LY2603618) | 107 | Cytarabine (U-19920A) |
| 52 | Trametinib (GSK1120212) | 109 | Methotrexate disodium |
| 53 | Ibrutinib (PCI-32765) | 109 | Methotrexate disodium |
|  |  | 111 | Erlotinib (OSI-774) HCl |

**Table S4. Statistical indicators of sample sequencing data**

| **sample** | **PDO_1** | **PDO_2** | **PDO_3** | **PDO_4** | **PDO_5** | **PT_3** | **PT_4** |
| --- | --- | --- | --- | --- | --- | --- | --- |
| [Total] Mapped Data (Mb) | 46989.82 | 46849.03 | 54846.55 | 46314.16 | 53789.2 | 57707 | 55521.39 |
| [Total] Fraction of Mapped Data (Mb) | 99.75% | 99.69% | 99.75% | 99.70% | 99.77% | 99.72% | 99.71% |
| [Total] PCR duplicate reads | 98913901 | 1010454141 | 17645285 | 103907484 | 136021227 | 121283700 | 11887979 |
| [Total] Fraction of PCR duplicate reads | 30.49% | 31.39% | 31.30% | 32.79% | 36.91% | 30.60% | 31.26% |
| [Target] Fraction of Target Reads in all reads | 72.98% | 72.53% | 70.11% | 71.66% | 72.15% | 70.84% | 70.77% |
| [Target] Average depth | 605.45 | 596.27 | 666.68 | 579.04 | 678.48 | 711.65 | 682.9 |
| [Target] Average depth (rmdup) | 408.37 | 397.18 | 444.5 | 377.56 | 415.22 | 480.65 | 455.81 |
| [Target] Coverage (>0x) | 99.78% | 99.70% | 99.91% | 99.89% | 99.59% | 99.91% | 99.90% |
| [Target] Coverage (>=4x) | 99.73% | 99.65% | 99.88% | 99.85% | 99.54% | 99.86% | 99.85% |
| [Target] Coverage (>=10x) | 99.62% | 99.58% | 99.80% | 99.78% | 99.46% | 99.79% | 99.77% |
| [Target] Coverage (>=30x) | 99.31% | 99.40% | 99.64% | 99.58% | 99.28% | 99.63% | 99.61% |
| [Target] Coverage (>=100x) | 97.75% | 98.34% | 98.84% | 98.43% | 98.15% | 98.80% | 98.76% |

**Table S5. Primer sequence**

| **Gene name** | **Primer sequence（5'→3'）** | |
| --- | --- | --- |
| N-cadherin | Forward Primer | TCAGGCGTCTGTAGAGGCTT |
|  | Reverse Primer | ATGCACATCCTTCGATAAGACTG |
| CDK4 | Forward Primer | ATGGCTACCTCTCGATATGAGC |
|  | Reverse Primer | CATTGGGGACTCTCACACTCT |
| CDK6 | Forward Primer | GCTGACCAGCAGTACGAATG |
|  | Reverse Primer | GCACACATCAAACAACCTGACC |
| CCNL1 | Forward Primer | TACCATCGACCACTCTCTGATT |
|  | Reverse Primer | GGATGCGTAAGTCCGTCTCAC |
| DCK | Forward Primer | CCATCGAAGGGAACATCGCT |
|  | Reverse Primer | GGTAAAAGACCATCGTTCAGGT |
| CD25 | Forward Primer | GTGGGGACTGCTCACGTTC |
|  | Reverse Primer | CCCGCTTTTTATTCTGCGGAA |
| CD69 | Forward Primer | ATTGTCCAGGCCAATACACATT |
|  | Reverse Primer | CCTCTCTACCTGCGTATCGTTTT |
| Gra B | Forward Primer | CCCTGGGAAAACACTCACACA |
|  | Reverse Primer | GCACAACTCAATGGTACTGTCG |
